# Noninvasive disruption of the blood-brain barrier in the marmoset monkey

**DOI:** 10.1038/s42003-023-05185-3

**Published:** 2023-08-02

**Authors:** T. Vincenza Parks, Diego Szuzupak, Sang-Ho Choi, Aydin Alikaya, Yongshan Mou, Afonso C. Silva, David J. Schaeffer

**Affiliations:** Department of Neurobiology, University of Pittsburgh, Pittsburgh, PA USA

**Keywords:** Blood-brain barrier, Neuroscience

## Abstract

The common marmoset monkey (*Callithrix jacchus*) is a species of rising prominence in the neurosciences due to its small size, ease of handling, fast breeding, and its shared functional and structural brain characteristics with Old World primates. With increasing attention on modeling human brain diseases in marmosets, understanding how to deliver therapeutic or neurotropic agents to the marmoset brain noninvasively is of great preclinical importance. In other species, including humans, transcranial focused ultrasound (tFUS) aided by intravenously injected microbubbles has proven to be a transient, reliable, and safe method for disrupting the blood-brain barrier (BBB), allowing the focal passage of therapeutic agents that do not otherwise readily traverse the tight endothelial junctions of the BBB. The critical gap that we address here is to document parameters to disrupt the BBB reliably and safely in marmosets using tFUS. By integrating our marmoset brain atlases and the use of a marmoset-specific stereotactic targeting system, we conduct a series of systematic transcranial sonication experiments in nine marmosets. We demonstrate the effects of center frequency, acoustic pressure, burst period, and duration, establish a minimum microbubble dose, estimate microbubble clearance time, and estimate the duration that the BBB remains open to passage. Successful BBB disruption is reported in vivo with MRI-based contrast agents, as well as Evans blue staining assessed ex vivo. Histology (Hematoxylin and Eosin staining) and immunohistochemistry indicate that the BBB can be safely and reliably opened with the parameters derived from these experiments. The series of experiments presented here establish methods for safely, reproducibly, and focally perturbing the BBB using tFUS in the common marmoset monkey that can serve as a basis for noninvasive delivery of therapeutic or neurotropic agents.

## Abstract (web summary)

A combination of MRI, histology, and immunohistochemistry is used to establish parameters to transiently and safely disrupt the blood-brain barrier in marmoset monkeys.

## Introduction

The focus of this manuscript is to establish parameters to safely disrupt the blood–brain barrier (BBB) in the common marmoset monkey (*Callithrix jacchus*), a species of rising prominence in the neurosciences. The BBB regulates the permeability of molecules to the brain parenchyma, consisting of capillary endothelium that prevents molecules with a weight of ~400 Da from entering^1^. In other preclinical modeling species (e.g., rats, mice, macaques, rabbits, pigs), transcranial focused ultrasound (tFUS) has become a reliable means to circumvent invasive intracerebral injections and allow for agent delivery by transiently disrupting the BBB^2–9^. The value of applying tFUS to noninvasively and locally disrupt the BBB in the marmoset model is potentially tremendous—for example, by taking advantage of the marmoset’s short interbirth interval and relatively short lifespan, tFUS can be used as a longitudinal and noninvasive method of neuromodulation^10^, neuronal tracing^11,12^, or even focal drug delivery^13^ for a disease model across the lifespan. With a lissencephalic cortex and cortical architecture that is more similar to humans than to rodents^14–17^, marmosets are ideal for tFUS, allowing for simplified targeting across the cortex when compared to the highly folded brains of other primate species.

The critical gap addressed here is to document the ability to reliably and noninvasively open the BBB in the marmoset using tFUS aided by microbubble cavitation. Microbubbles are microscopic (~1–10 μm) gas-filled micelles that can be systemically injected just before ultrasonic stimulation^18^. At lower acoustic pressures, microbubbles oscillate (stable cavitation) and when exposed to sufficient pressure can collapse (inertial cavitation) and release a powerful liquid jet through the endothelium that can potentiate agent delivery^18–20^. With myriad available neuromodulatory or therapeutic agents having been shown to cross the BBB as a result of ultrasound-mediated microbubble cavitation^13,21–23^, understanding the parameters to open the BBB in the marmoset will inevitably lead to numerous neuroscientific applications. We expect this technique will have broad application in translational marmoset research, especially for neurodevelopmental applications, providing a means to track the neuropathological emergence of circuit dysfunction noninvasively from a young age.

**Fig. 1:**
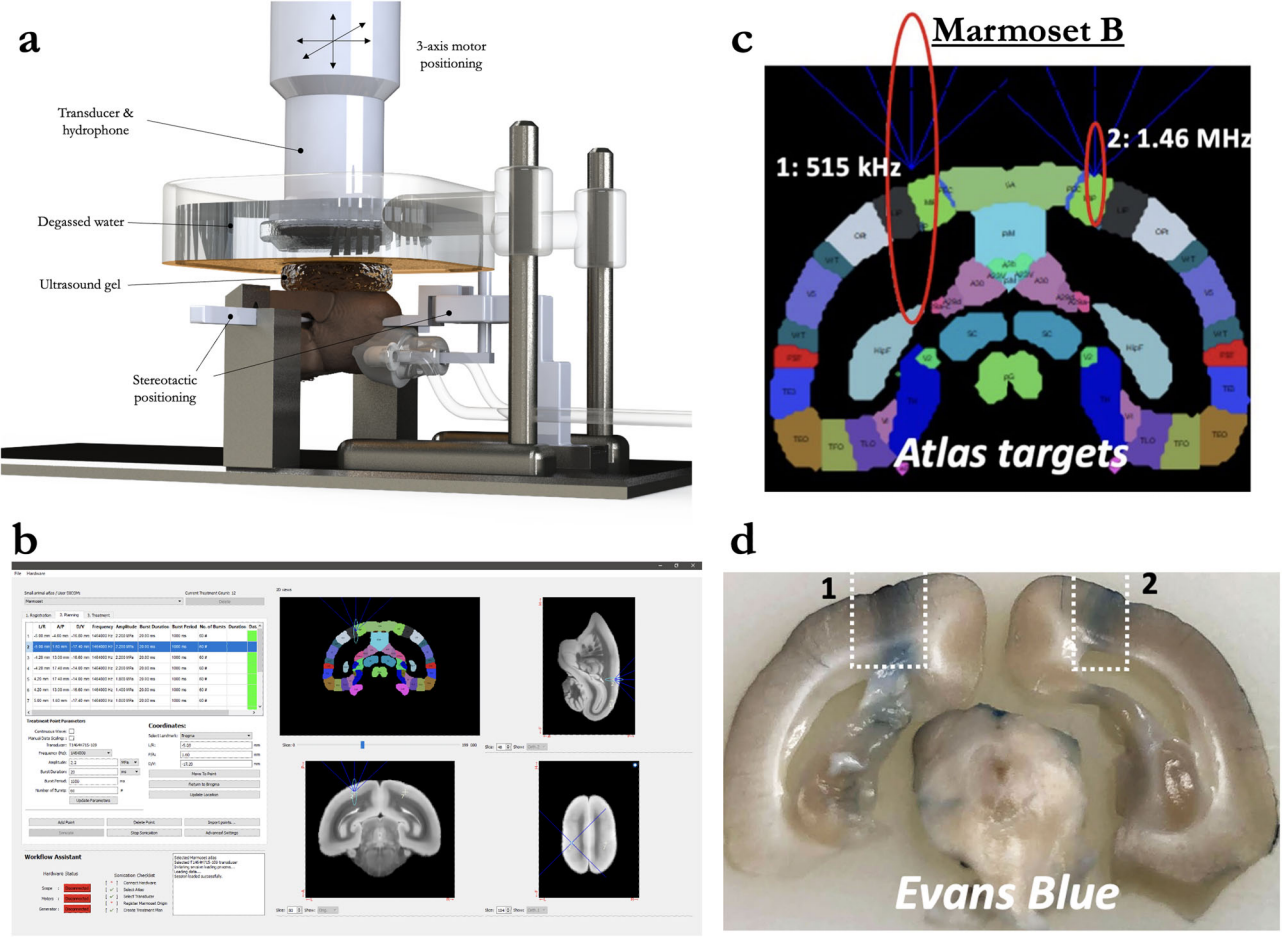
Focused ultrasound positioning apparatus, customized for use in marmoset monkeys. **a** Computer-aided design three-dimensional rendering of the stereotactic positioning of the marmoset with reference to the transducer and hydrophone. **b** Software MORPHEUS (FUS Instruments, Toronto, Ontario, Canada) with our marmoset atlases integrated along with cytoarchitectonic boundaries. **c** Relative size of BBB disruption using 515 kHz and 1.46 MHz transducer overlayed on marmoset atlas. **d** Relative size of BBB disruption as reported by Evans blue staining in Marmoset B.

Through the combination of a marmoset-specific atlas (cytoarchitectonic boundaries registered^24^ to MRI space^25,26^) and a now commercially available marmoset-specific focused ultrasound apparatus (Fig. 1; RK-50 Marmoset, FUS Instruments Incorporated, Toronto, ON, Canada), we demonstrate the requisite parameters to reliably stereotactically target and open the BBB to a small parenchymal volume (~10–20 mm^3^) with a single element 1.46 MHz transducer. We demonstrate the effects of center frequency on BBB disruption size, acoustic attenuation due the marmoset skull, the minimum acoustic pressure to disrupt the BBB, and the deleterious effects of too much acoustic pressure. We also demonstrate the minimum microbubble dosage for BBB disruption at safe acoustic pressure, estimate microbubble clearance time, and the effect of skull angle on BBB disruption. Through reporting by in vivo gadolinium-enhanced MRI and ex vivo Evans Blue staining as well as histological and immunohistochemical reporting, we provide a detailed account of the requisite parameters for safely, reproducibly, and focally disrupting the BBB in the common marmoset.

## Results

### Assessment of performance and accuracy

Based on the CT imaging of the melted acrylic test plate, we quantified the Euclidean distance from each sonication point to the target location. Supplementary Fig. 1g shows the accuracy across the 24 individual sonication points across the XY plane. The average spatial error was 220 μm in X, 117 μm in Y, and 247 μm in the Z direction.

### BBB disruption as a function of center frequency

Marmoset B received two sonications in parietal cortex to determine the relative difference in BBB disruption size as a function of transducer center frequency. With the goal of minimizing the size of disruption, both transducers (515 kHz, left hemisphere; 1.46 MHz, right hemisphere) were focused on the edge of the cortex (Fig. 1c)—consequently, only about half of the ultrasonic beam in the axial orientation was focused on brain tissue. As evidenced by the Evans blue staining (Fig. 1d), both sonications were close in volume to the full width at half maximum of the acoustic pressure distribution at the focus (with 515 kHz at 188 mm^3^ and 1.46 MHz at 10 mm^3^). With the aim of subsequent experiments to demonstrate focal BBB disruptions, only the higher-frequency 1.46 MHz transducer was used. As reliably demonstrated across the experiments described below, the method of centering the focus of the 1.46 MHz transducer at the top of the cortex allowed for disruptions on the order of 1 mm radially, and 2.5 mm axially. Indeed, the disruption size varied slightly as a function of acoustic pressure, microbubble dosage, burst duration, and number of bursts, but at the “safe” parameters demonstrated below, the 1.46 MHz transducer allowed for cortical disruptions within the bounds of most cortical cytoarchitectonic regions of interest (e.g., fit within the medial-lateral extent of area MIP).

### BBB disruption as a function of acoustic pressure

**Fig. 2:**
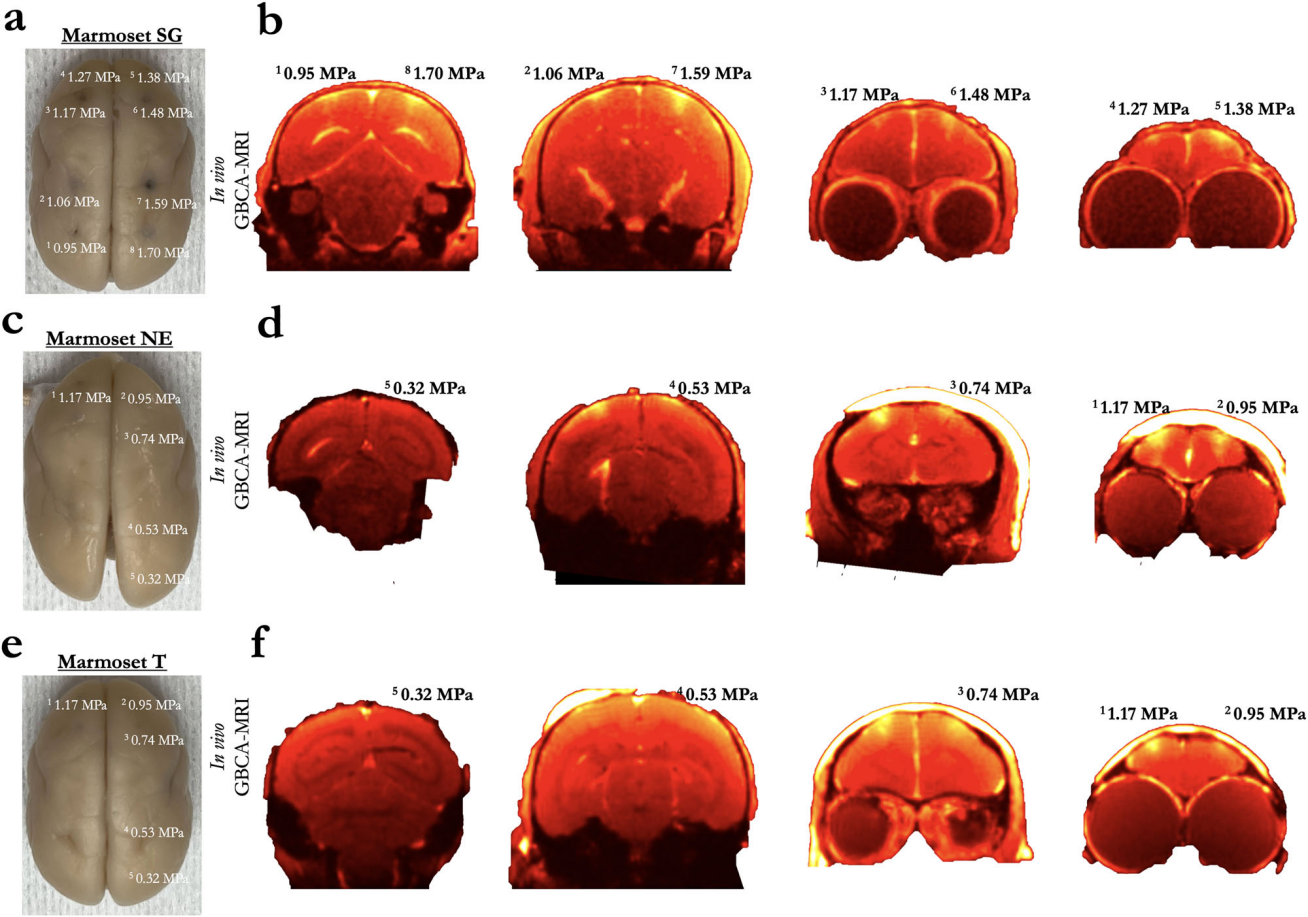
Blood–brain barrier disruption as a function of derated acoustic pressure, minimum pressure. **a** Resultant disruption of the BBB at eight sonications across the cortex of marmoset SG shown via extravasation of Evans blue in Marmoset SG**. b** Extravasation of GBCA using in vivo MRI in Marmoset SG. **c**, **d** Same as (**a**, **b**) in Marmoset NE who was sonicated at lower acoustic pressures. **e**, **f** Same as (**c**, **d**) in Marmoset T.

Marmosets SG, NE, and T each received five or eight cortical sonications (area 8a, 4ab, MIP, and V2) with a 1.46 MHz transducer. Figure 2 shows the sonication locations and accompanying acoustic pressures. Based on the initial experiments in marmoset SG demonstrating that derated acoustic pressures of 0.95–1.70 MPa (and a high 400 μl/kg microbubble dose) allowed for reliable BBB disruption, marmosets NE and T were sonicated with lower derated pressures of 0.32–1.17 MPa and a lower 200 μl/kg microbubble dose to determine the minimum pressures at which perturbing the BBB allows for extravasation of GBCA and Evans blue stain. As shown in Fig. 2, 0.53 MPa (derated) perturbed the BBB in marmoset NE, but not marmoset T. 0.74 MPa (derated) successfully perturbed the BBB in both marmoset NE and T, but there was a clear superficial cortical bias of the distribution of BBB disruption closer to the center of the focus of the acoustic beam. At a derated pressure of 0.95 MPa, marmoset NE showed a clear perturbation at a size approximating volume to the full width at half maximum of the acoustic pressure distribution at the focus (2.5 mm axially by 1 mm radially), but less so in marmoset T. At 1.17 MPa (derated) marmoset SG, NE, and T showed a consistent disruption (note that the GBCA agent dosage for marmoset SG was lower at 0.1 ml/kg, but see photograph of Evans blue staining in Fig. 2 at 1.17 MPa).

### Minimum microbubble dosage to disrupt the BBB

**Fig. 3:**
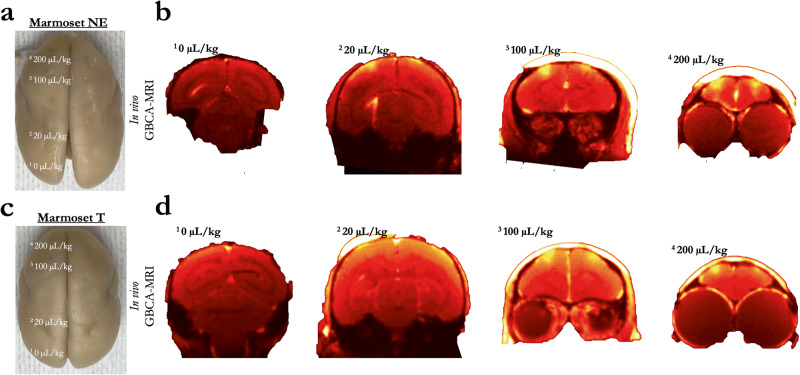
Minimum microbubble dosage for reliable blood–brain barrier disruption. **a** Resultant disruption of the BBB at four sonications with varied microbubble dosages in Marmoset NE. **b** Extravasation of GBCA using in vivo MRI in Marmoset NE. **c**, **d** Same as (**a**, **b**) in Marmoset T.

The right hemispheres of marmosets NE and T were used to determine the minimum acoustic pressure (Fig. 2), while the left hemisphere was used to demonstrate the minimum microbubble dosage necessary to perturb the BBB (Fig. 3). Each animal was sonicated four times (area 8a, 4ab, MIP, and V2), with the lowest microbubble dosage used for the most posterior sites from 0 μl/kg (V2), then increasing to 20 μl/kg (MIP), 100 μl/kg (4ab), and 200 μl/kg (8a) at the anterior sites. Though the parameters (and timing) were otherwise the same for marmosets NE and T, 20 μl/kg perturbed the BBB in marmoset NE, but not fully in marmoset T. In total, 100 μl/kg successful perturbed the BBB in both animals, as did 200 μl/kg. Thus, with our apparatus, we demonstrate minimum microbubble dosage for single-bolus tail vein injections in marmosets to be in the range of 20–100 μl/kg at 1.46 MHz.

### Microbubble clearance

**Fig. 4:**
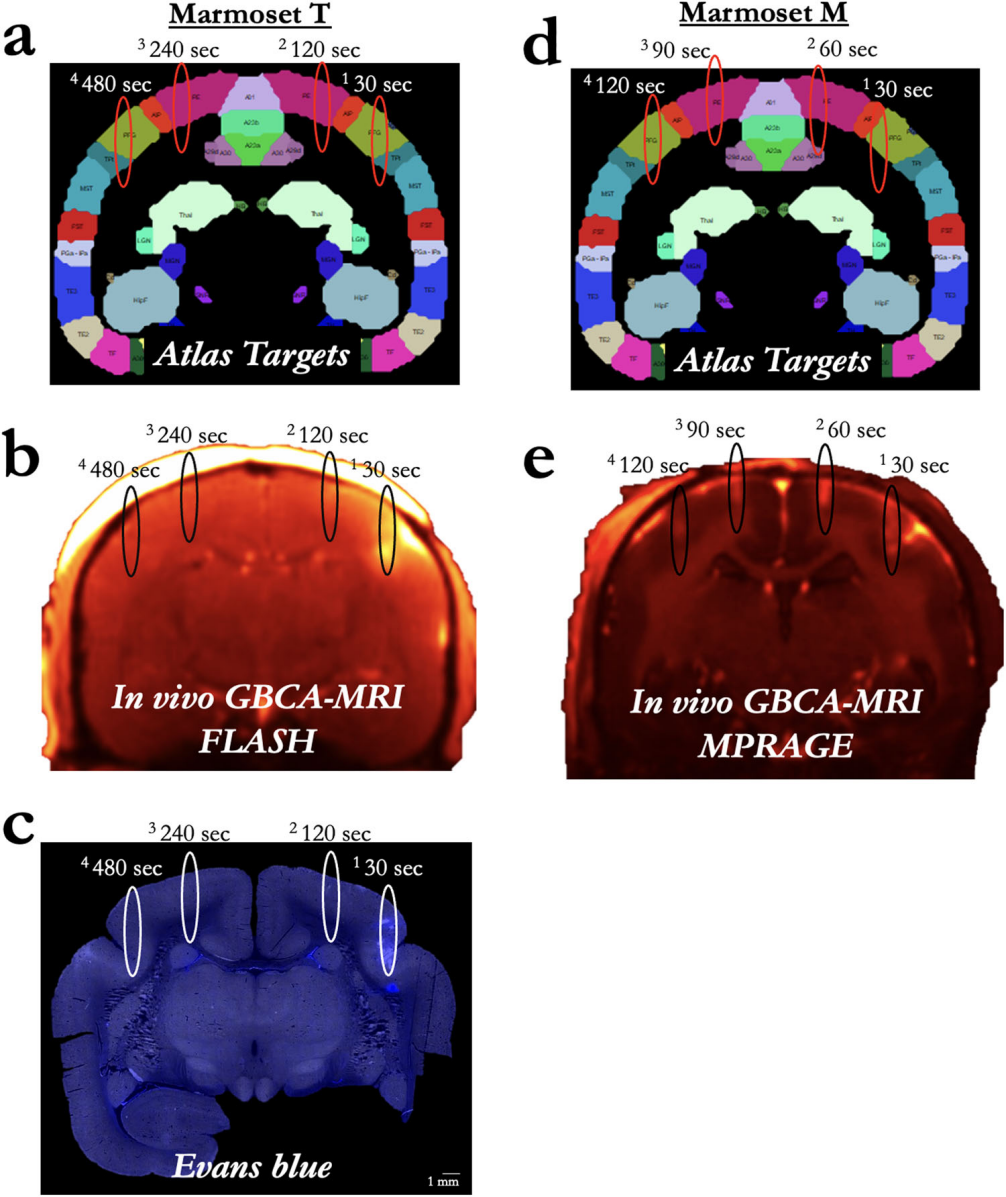
Microbubble clearance. **a** Sonication target locations for Marmoset T at 30–480 s after a single bolus injection of 200 µl/kg of microbubbles. **b** GBCA contrast enhancement due to successful BBB disruption in Marmoset T. **c** Sites in Marmoset T as reported by Evans blue staining. **d** Sonication target locations for Marmoset M, but at 30–120 s after a single bolus injection of microbubbles at 200 µl/kg. **e** Same as (**b**), for Marmoset M. Note: the MRI sequence varied for Marmosets T and M due to Marmoset M participating in other experiments, but both clearly showed T1-weighted GBCA contrast. No Evans blue was injected in Marmoset M.

With a total of 12 sonications (8 of which are described in the previous two sections), Marmoset T also received four sonications (parietal areas PFG right, PE right, PE left, and PFG left) to determine the ability to disrupt the BBB across multiple sites after a single bolus injection of microbubbles. Figure 4 shows sonication locations 30, 120, 240, and 480 s after a single bolus injection of 200 μl/kg of microbubbles; each of the four sonications had the following parameters: derated acoustic pressure = 1.17 MPa, burst duration = 20 ms, burst period = 1000 ms, number of bursts = 60. At 1.17 MPa, only the sonication 30 s after microbubble injection showed perturbation which allowed for clear extravasation of GBCA and Evans blue stain at the approximate volume at FWHM of the acoustic pressure distribution at the focus, as visualized by the ellipsoids in Fig. 4a–e. Marmoset M shared the same sonication parameters and was used to determine the ability to disrupt the BBB at 30, 60, 90, and 120 s (between the first two points of Marmoset T) after a single bolus injection. From this data, the effective clearance time of a 200 μl/kg dose of commercially available microbubbles (Definity) appears to be less than 2 min in the marmoset brain—we expect that there is some variation in effective concertation of intravascular microbubbles as a function of the animal’s physiological state (e.g., heart rate, kidney function). The dose here, however, was relatively high at 200 μl/kg, so the availability of bubbles in circulation could not be much higher without leading to damage. Across the (other) experiments presented here, we injected prior to each sonication rather than using a single bolus or infusion for multiple sonications, with at least 5 min between injections. Likely due to variation in tissue (e.g., muscle, fat) between the sonicated site and the hydrophone, we did not find the Fourier spectrum of the monitored cavitation emissions to be a particularly reliable index of BBB perturbation (with ground truth from the Evans blue staining microscopy). However, this experiment allowed for the opportunity of comparing spectra from successful disruptions to unsuccessful ones with identical parameters, only varying available microbubbles in circulation. From these four sonications (marmoset T), broadband noise and an increase in subharmonic amplitude around half of the transducer frequency (0.73 MHz) appeared in relation to cavitation corresponding to disruption at 30 and 120 s post microbubble injection, but only the 30 s post-microbubble injection sonication showed successful BBB opening (Supplementary Fig. 2).

### BBB disruption as a function of skull angle

**Fig. 5:**
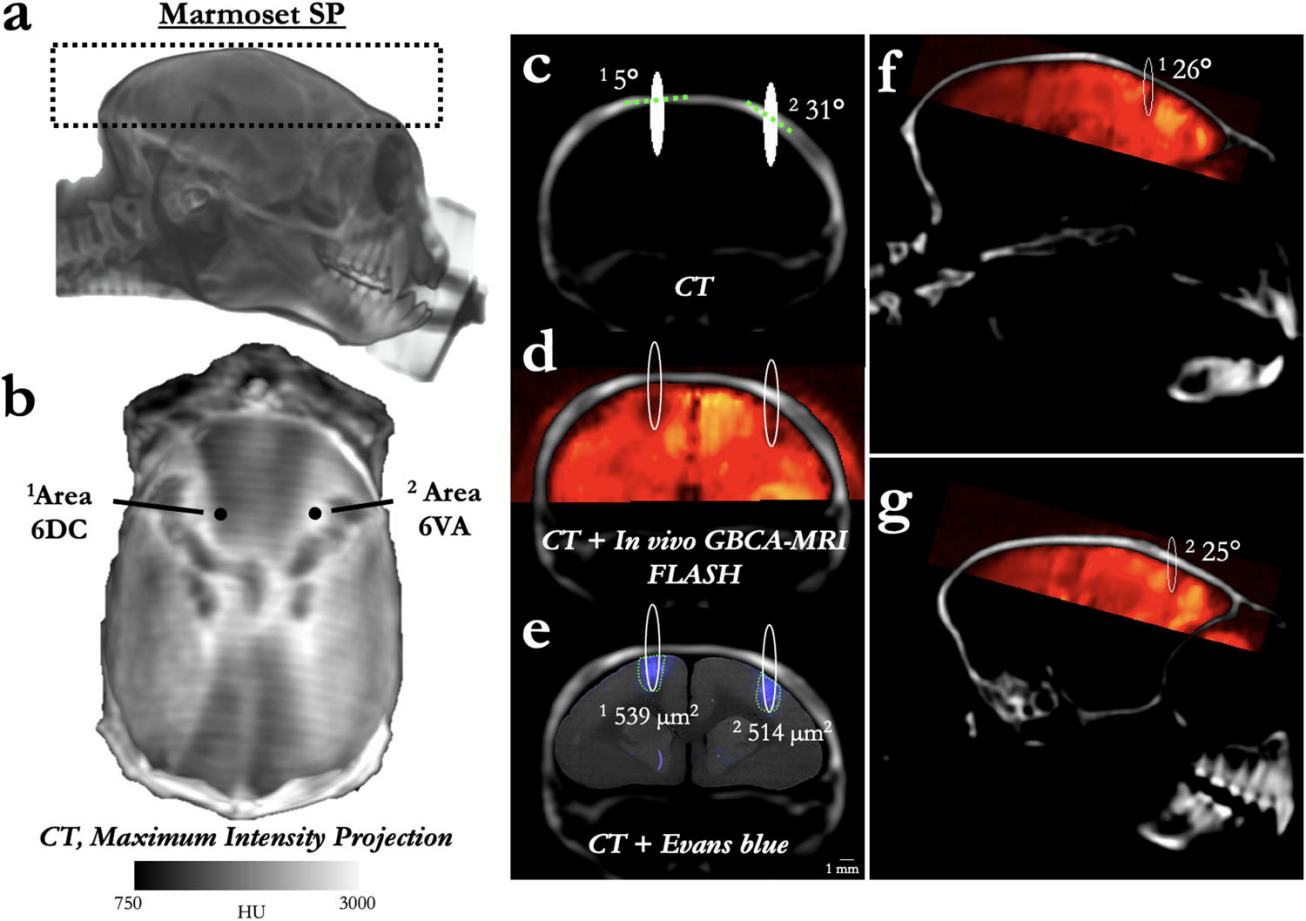
BBB disruption across varied skull thickness and angle. **a** Three-dimensional rendering of Marmoset SP’s CT, with the black dashed lines showing the window for the maximum intensity projection (MIP) image shown in (**b**). **b** Distribution of density (as indexed by Hounsfield Units (HU)) across Marmoset SP’s skull with reference to sonication locations 1 and 2—other than location, sonication locations 1 and 2 share the same parameters and microbubble dosage. **c** Coronal slice of Marmoset SP’s skull with the sonication location overlaid (FWHM of the acoustic pressure distribution at the focus) and the skull angle (dashed green line tangent to skull). Note that the opening appears hypo- rather than hyper-intense because of the high GBCA dosage used in this animal. **d** Resultant opening with in vivo GBCA-MRI aligned to the same animal’s CT. **e** Despite the varied thickness and skull angle, the BBB opening size was minimally affected, with Evans blue staining (microscopy image aligned to CT) showing an opening volume of sonication site 2 to be 95% of that of sonication site 1. **f**, **g** Same images as in (**d**), but in sagittal slices with green dashed line showing the skull angle in that place.

**Fig. 6:**
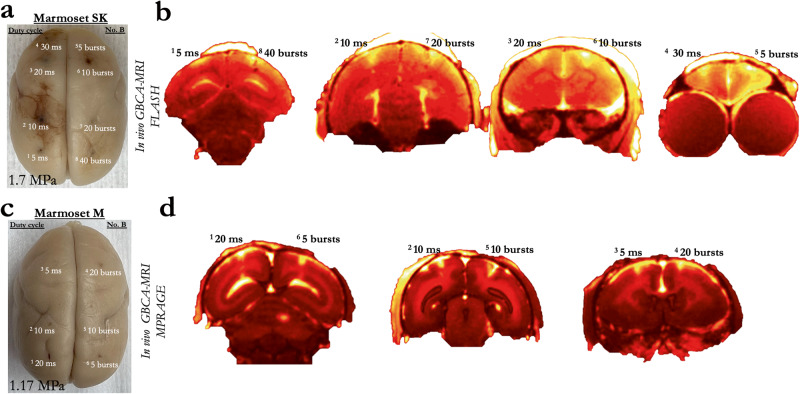
Blood–brain barrier disruption as a function of burst duration and number of bursts. **a** Extravasation of Evans blue showing the resultant disruption of the BBB at the site of eight sonications, with varied duty cycle and number of bursts in Marmoset SK at 1.7 MPa. **b** Extravasation of GBCA using in vivo MRI in Marmoset SK. **c** Same as (**a**), for Marmoset M at a lower acoustic pressure of 1.17 MPa. **d** Same as (**b**), for Marmoset M. Note that the MRI sequence varied for Marmosets SK and M due to Marmoset M participating in other experiments, but both clearly showed T1-weighted GBCA contrast.

**Fig. 7:**
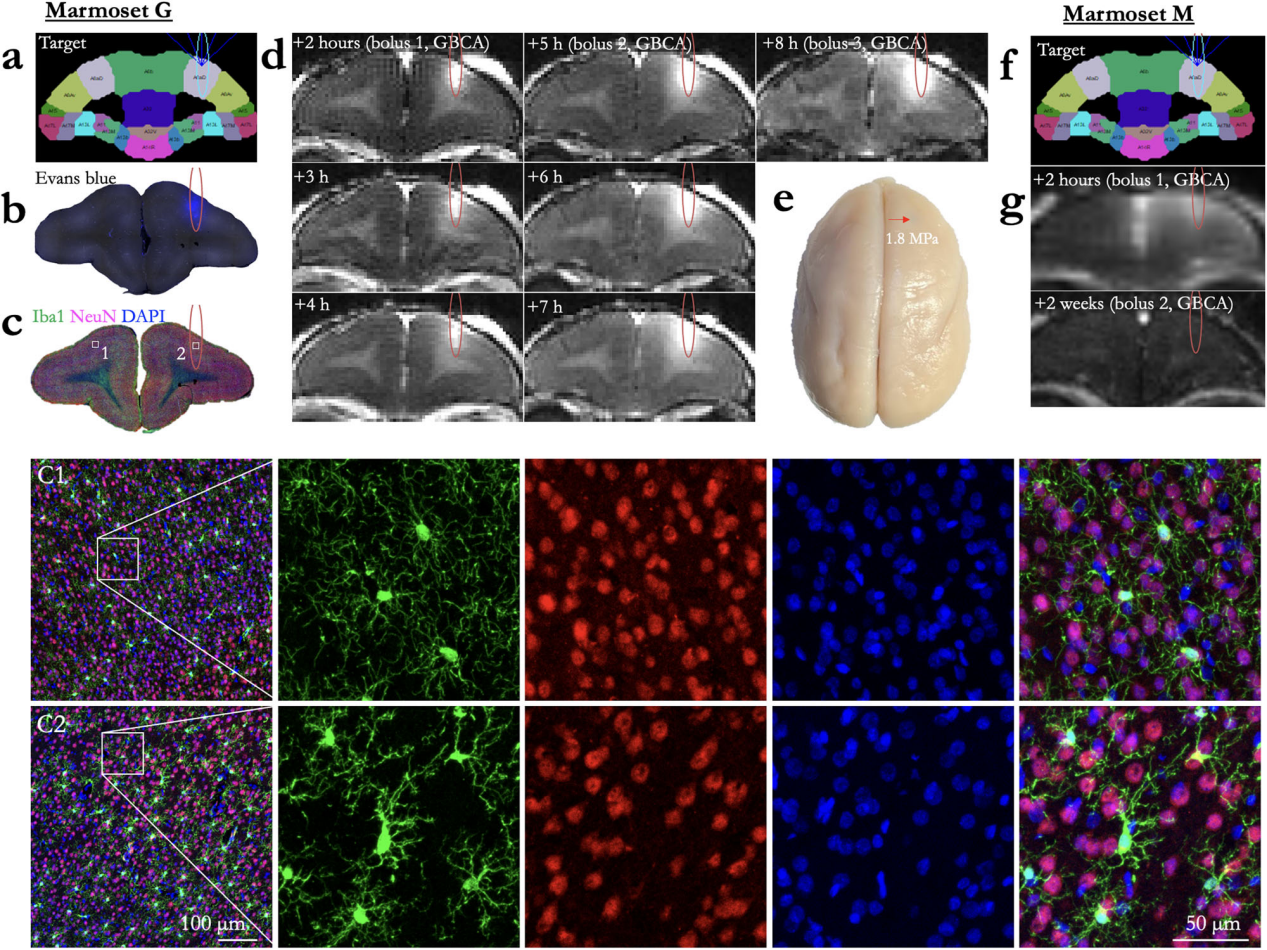
Blood–brain barrier opening duration and immunohistochemistry. **a** Target for a single sonication in Marmoset G, area 8a. **b** Extravasation of Evans blue as the result of the sonication in (**a**). **c** Immunostaining at the sonicated slice, with C1 showing the left (not sonicated) hemisphere and C2 showing right hemisphere within the bounds of the sonicated area. Compared to left hemisphere, a marked increase in Iba1+ cells was clearly evident in right hemisphere. Iba1+ cells exhibit larger cell bodies and thick processes in right hemisphere, whereas Iba1+ cells show much more numerous and finer processes in left hemisphere. **d** In vivo GBCA MRI, with boluses of GBCA injected at 2, 5, and 8 h after sonication. As shown in the hours following the bolus injections (hours 3 and 4, also hours 6 and 7), the size of the GBCA remained the same (i.e., minimal diffusion), but increased with subsequent bolus injections at hours 5 and 8, suggesting the BBB was still open at 8 h post sonication. **e** Marmoset G’s perfused brain, sonication site marked by a red arrow, indicating no evidence of hemorrhage at this site. **f** Target for a single sonication in Marmoset M, area 8a. **g** Extravasation of GBCA, injected as a bolus 2 h after sonication, then a second bolus injection again after 2 weeks. After 2 weeks, the BBB was no longer permeable to the GBCA.

With known effects of acoustic reflection mediated by skull angle and thickness^27^, we sought to directly test these effects by sonicating at 1.46 MHz across varied skull angles of the marmoset head. As shown in the apparatus rendering in Fig. 1, our transducer was always parallel with the stereotactic plane (i.e., the Z plane corresponding to the center of the ear bars and bottom of the eye bars) and thus was fixed with reference to the varying skull angle. In addition to in vivo GBCA-enhanced MRI, high-resolution (50 μm isotropic) CT images were acquired in marmoset SP to isolate the skull and accurately measure angles tangent to the skull surface (tangent to the point at the intersection of the axial center of the sonication ellipsoid) (Fig. 5). At high (derated) pressure (1.70 MPa, 400 μl/kg microbubble dose), the size of the BBB disruption matched the FWHM of the expected sonication distribution, indicating the sonications planar to (above the) marmoset skull were not adversely affected by skull angle. Accordingly, the thin marmoset skull (~1 mm or less) seems to be amenable to focused ultrasound compared with other species, with 47% deration measured directly through marmoset skulls here. It is also worth noting that although the acoustic pressure and microbubble dose were high for this experiment, data from Figs. 2, 3, 6 or 7 also demonstrate that skull angle seems to have a minimal effect at lower acoustic pressures and microbubble dosages. Because of the focus of our transducer (focal length = 24.5 mm), we have yet to establish these effects deeper within the brain. With the transducer used here, we could only effectively center the acoustic beam 11 mm from the top of the marmoset head, limiting the ability to establish such effects.

### BBB disruption as a function of burst duration and number of bursts

Marmosets SK received 8 sonications and Marmoset M received 6 sonications to determine the effect burst duration and number of bursts on the extent of BBB disruption—and, although unintentional, the extent and nature of damage at high acoustic pressure for Marmoset SK (Fig. 6). Indeed, for marmoset SK, with such a high pressure and microbubble dosage, all the tested duty cycles (5, 10, 20, and 30 ms) and number of bursts (5, 10, 20, 40) disrupted the BBB in marmosets SK. These data, however, are particularly useful for demonstrating the extent of damage that can occur at the extremes of acoustic pressure, burst durations, and at high microbubble dosages (400 μl/kg), as shown in Supplementary Fig. 3. For Marmoset M, however, the derated pressure (1.17 MPa) and microbubble dosage (200 μl/kg) were closer to the boundary of safe sonication parameters. Of the tested parameters, as low as 5 bursts (20 ms burst duration) or 5 ms burst duration (60 bursts), we found that we were likely above the minimum to disrupt the BBB. These data suggest that the disruption likely occurs with the first few bursts, when the circulating microbubble concertation is highest. This bodes particularly well for applications in which very fast sonications are advantageous, such as those delivered while the animal is awake and performing a task.

### BBB disruption duration

**Fig. 8:**
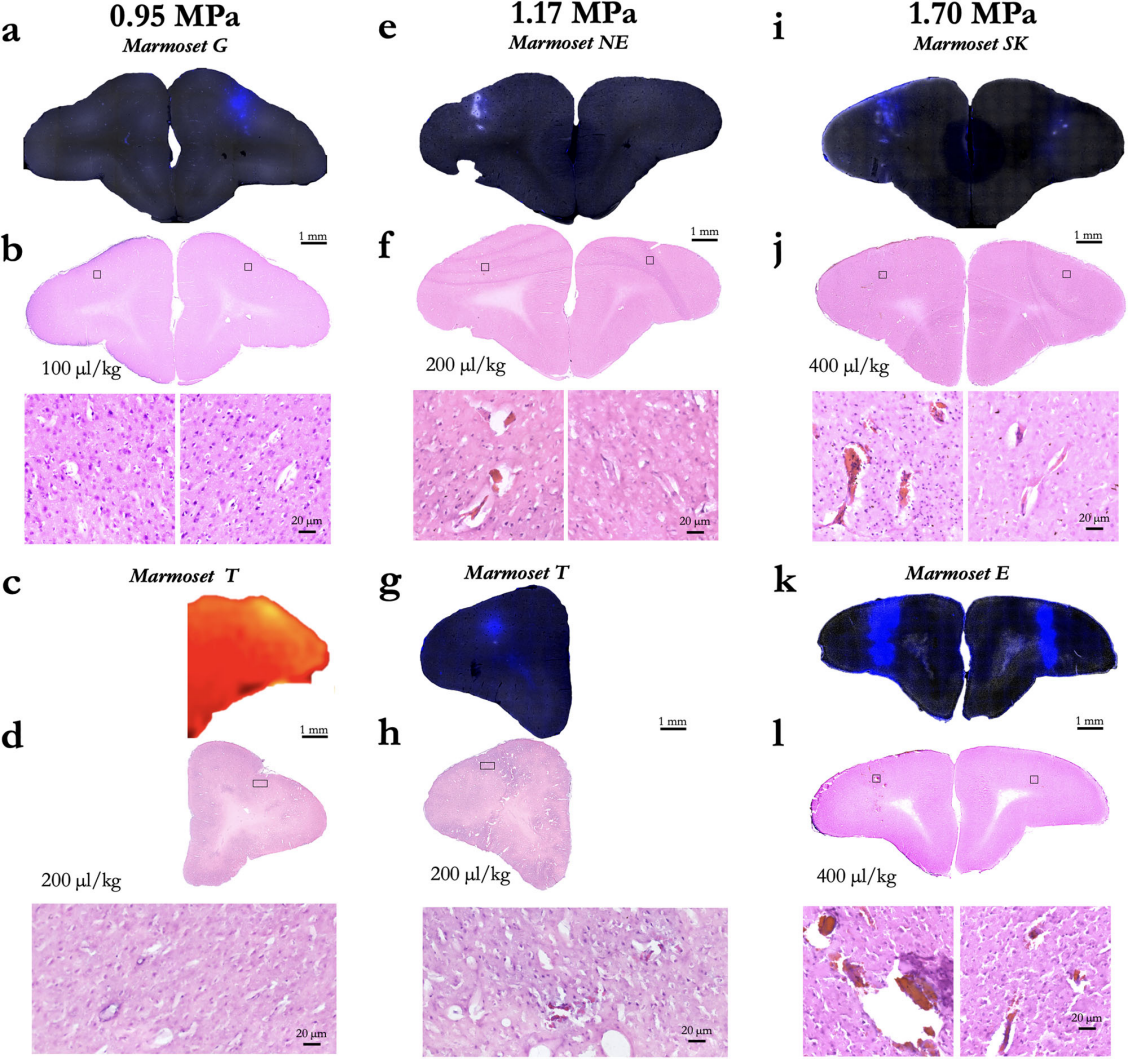
Cortical damage as a function of acoustic pressure and microbubble dosage. **a** Evans blue microscopy image of sonication at 0.95 MPa with 100 µl/kg microbubble dose in Marmoset G. **b** H&E stained slice of interest from (**a**). Black boxes overlaying the H&E images show the location of the zoomed image below. **c** GBCA image of sonication at 0.95 MPa with 0.95 MPa with 200 µl/kg microbubble dose in Marmoset T. **d** H&E for (**c**). **e** Same as (**a**), using 1.17 MPa with 200 µl/kg microbubble dose in Marmoset NE. **f** H&E for (**e**). **g** Same as (**a**), Marmoset T: 1.17 MPa, 200 µl/kg microbubble dose. **h** H&E for (**g**). **i** Same as (**a**), Marmoset SK: 1.7 MPa, 400 µl/kg microbubble dose. **j** H&E for (**h**). **k** Same as (**a**), Marmoset E: 1.7 MPa, 400 µl/kg microbubble dose. **l** H&E for (**k**).

**Fig. 9:**
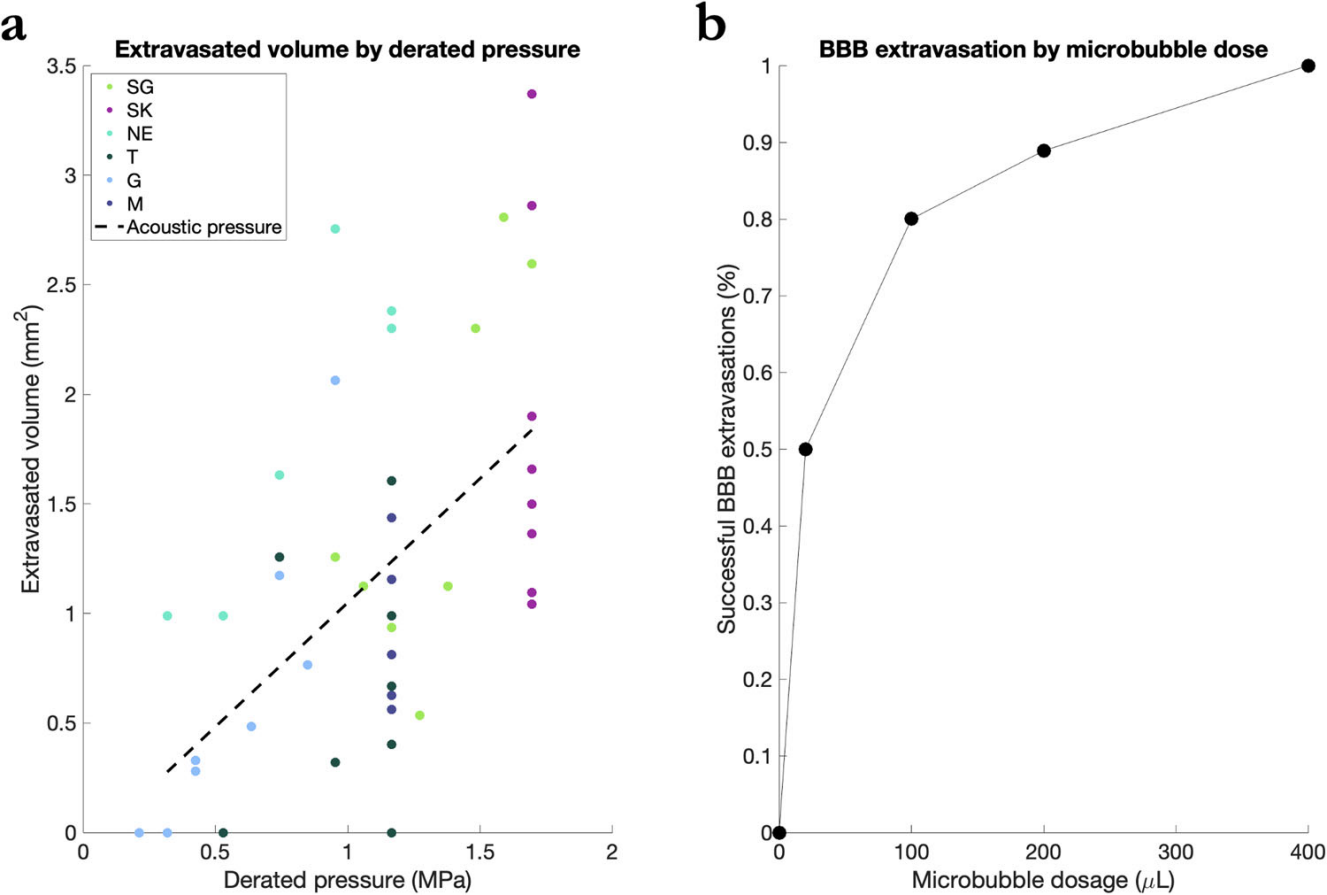
BBB extravasation by derated pressure and microbubble dose. **a** Volume of GBCA extravasated into brain parenchyma as a function of derated acoustic pressure. Different marmosets are indicated by dot color. Dashed line shows fit of linear regression accounting for variance due to derated pressure. **b** Successful BBB extravasation of GBCA as a function of microbubble dosage, irrespective of other parameters.

Marmoset G received a single sonication (area 8a, right hemisphere) with a 1.46 MHz transducer and the following parameters: derated acoustic pressure = 0.95 MPa, burst duration = 20 ms, burst period = 1000 ms, number of bursts = 60, microbubble dosage = 100 μl/kg. As shown in Fig. 7, BBB disruption was reported by in vivo GBCA-enhanced MRI and Evan’s blue staining. From the previous experiments and the results shown in Figs. 7–9, we found these parameters to be both safe and effective at opening the BBB. In addition to demonstrating the safety and reliability of opening with the aforementioned parameters, we sought to determine the length of time that BBB remained open. As this was a scheduled terminal experiment (with Evans blue already injected, precluding recovery of the animal), we were not able to determine the upper limit BBB disruption duration. Rather, our last point of imaging was 8 h after injection. As shown in Fig. 7, the BBB was clearly still open at 8 h post-sonication as indicated by increased intensity and distribution resulting from the GBCA and at the start of the 8-h post-sonication scan. Marmoset M, however, was not injected with Evans blue and was injected with GBCA 2 weeks after sonication—the BBB no longer allowed passage of a sufficient volume of GBCA to be detected. Note that the images from Fig. 6 were acquired at the same time as Marmoset M’s image in Fig. 7 (demonstrating that the GBCA injection was successful), with extravasation of GBCA at the sites sonicated 2 weeks after the first 8a sonication.

### Quantification of extravasated volume across the BBB

To determine what parameters accounted for the most variance in successful extravasation of GBCA across the BBB, a linear regression analysis was performed, and the data is visualized in Fig. 9a; source data found in Supplementary Data 1. The results of the regression indicated two predictors explained 38.2% of the variance (*R*^2^ = 0.38, *F*(42) = 5.2, *p* < 0.00). Derated acoustic pressure significantly predicted extravasated volume (*β* = 0.83, *p* < 0.02), while microbubble dose, number of bursts, burst periods, and animal weight did not significantly predict volume. When the same model was computed with stepwise regression, the derated pressure alone predicted 31.1% of the variance (*R*^2^ = 0.31, *F*(46) = 20.7, *β* = 1.13, *p* < 0.00). Microbubble dosage, however, was significantly related to whether a BBB opening was achieved as quantified by a linear regression with a binarized dependent variable (BBB perturbation or not). The results indicated two predictors explained 31.3% of the variance (*R*^2^ = 0.31, *F*(42) = 3.7): microbubble dose (*β* = 0.00, *p* = 0.01) and animal weight (*β* = 4.62, *p* = 0.02). Further, regardless of other parameters, BBB perturbation was achieved in 50% of sonications using 20 µl dosage, 80% of sonications using 100 µl, 89% using 200 µl, and 100% using 400 µl (see Fig. 9b).

#### Histology and immunohistochemistry

To determine efficiency and safety of BBB disruption according to changes in acoustic pressure and microbubble dosage, both Evans blue extravasation and histological tissue damage were confirmed in response to a derated acoustic pressure of 0.95, 1.17, and 1.70 MPa with 100, 200, or 400 μl/kg microbubble dosage. As shown in Fig. 8, Evans blue extravasation was observed for a derated acoustic pressure of 0.95 MPa and higher acoustic pressure (1.17 and 1.70 MPa). Acoustic pressure of 1.70 MPa with 400 μl/kg microbubble dosage produced severe tissue damage and large clusters of erythrocyte extravasation in marmoset SK, whereas an acoustic pressure of 1.17 MPa facilitated BBB disruption in the absence of apparent tissue damage or microhemorrhages in marmoset G and T. Moreover, there was no apparent neuronal damage identifiable in this region when examining the NeuN immunostaining (Fig. 7). Ionized calcium-binding adapter molecule 1 (Iba1) expression is upregulated upon microglia activation. Therefore, we used immunostaining of this microglial marker as measures of immune response. Compared to contralateral hemisphere, a marked increase in Iba1^+^ microglia was clearly evident in ipsilateral hemisphere (Fig. 7). Iba1^+^ cells exhibited larger cell bodies and thick processes in ipsilateral hemisphere, whereas Iba1^+^ cells showed much more numerous and finer processes in the contralateral hemisphere.

**Fig. 10:**
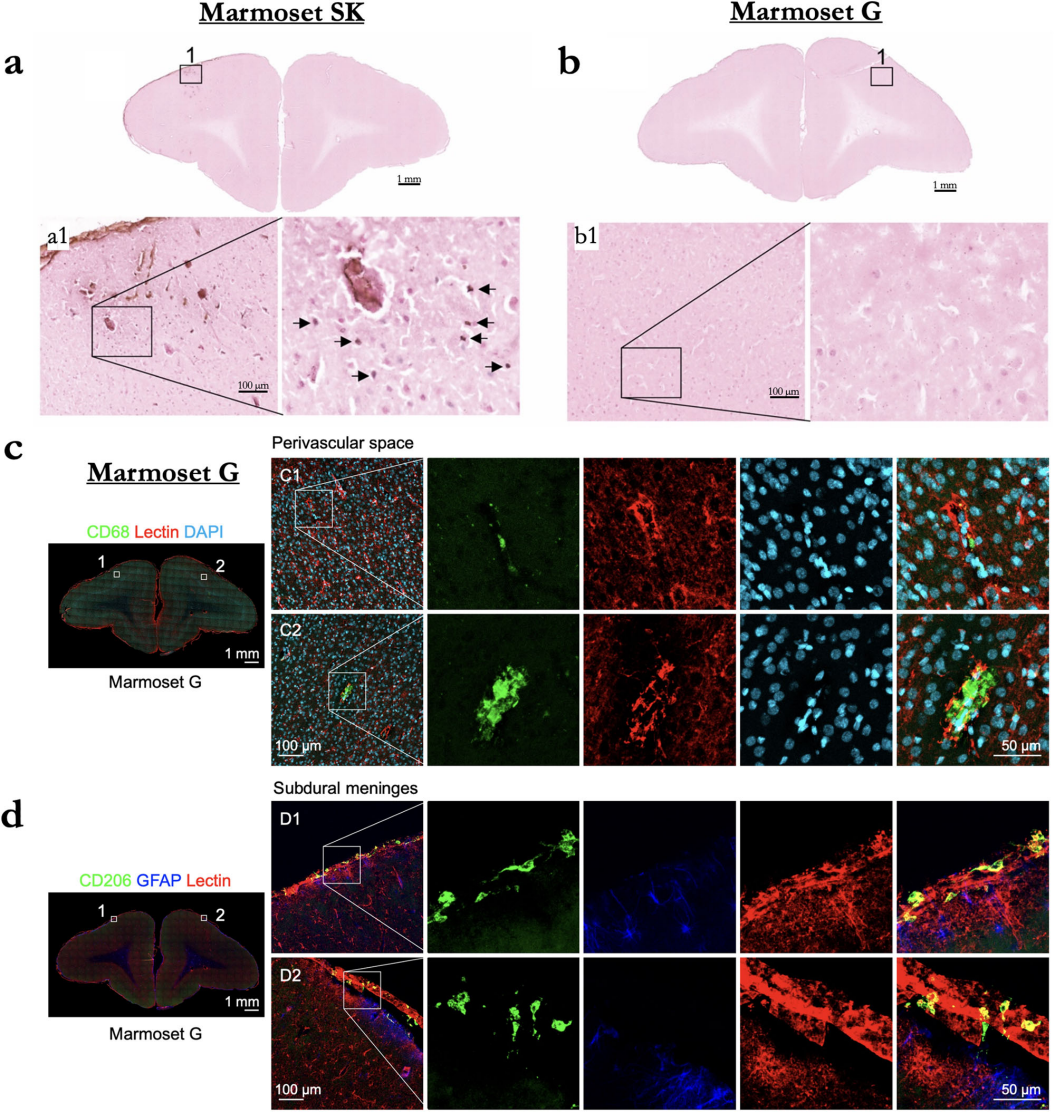
Analysis of cellular damage by TUNEL staining in Marmoset SK and G. **a** TUNEL-positive cells in brain tissue (arrows) in Marmoset SK. **b** TUNEL-positive cells in Marmoset G. **c** CD68 expression in Marmoset G was localized to the perivascular space. **d** Coronal brain sections depicting subdural meningeal endothelial cells (tomato lectin, red) and macrophages (CD206, green).

As a further assessment of safety, histological tissue damage was confirmed in response to derated acoustic pressures of 0.95 and 1.70 MPa with 100 or 400 μl/kg microbubble dosage (Fig. 10). Acoustic pressure of 1.70 MPa with 400 μl/kg microbubble dosage produced severe tissue damage and TUNEL-positive cells in Marmoset SK, whereas an acoustic pressure 0.95 MPa facilitated BBB disruption in the absence of apparent tissue damage or TUNEL-positive cells in Marmoset G. Further, we performed additional immunofluorescence staining in Marmoset G using two different antibodies (CD68 and CD206). Microglia and border-associated macrophages could not be unambiguously distinguished because they share common microglia/macrophage markers. We were able to identify the presence of presumably activated Iba1^+^ microglia with shorter and thicker processes as compared to the normal surveilling and more ramified microglia present in the contralateral hemisphere. CD68 is a common marker for macrophage lineage, primarily localized to microglia within the brain parenchyma, and perivascular macrophages in the cerebral blood vessels, and occasionally, parenchyma. Although, there is some CD68 expression on resting microglia, it labels the lysosome and is therefore commonly considered a marker of activated phagocytic microglia. CD206 is a cell-surface protein abundantly presents on selected populations of macrophages. There was no notable change observed in the numbers of CD68^+^ or CD206^+^ cells in the cortex of Marmoset G (Fig. 10). There were sparse CD68^+^ and CD206^+^ macrophages localized in perivascular space and subdural meninges. Therefore, the major immune-related cell type observed in the ipsilateral hemisphere was resident microglia.

## Discussion

In this study, we sought to establish the parameters to focally disrupt the BBB across a cohort of marmoset monkeys. By integrating our MRI-based marmoset atlases^25,26^ with a motorized stereotactic positioning system (RK-50 Marmoset; FUS Instruments Incorporated, Toronto, ON, Canada, Fig. 1) we were able to focally sonicate sites across the dorsal surface of the marmoset cortex with high accuracy (Figs. 1–8 and Supplementary Fig. 1) by way of spherically focused single-element transducers. Of the two transducers tested—at 515 kHz and 1.46 MHz—we found that the higher frequency 1.46 MHz transducer (with a 24.5 mm focal length) allowed for disruptions that could be limited to the ~2.5–3 mm cortical thickness of the marmoset cortex. We optimized parameters (minimum acoustic pressure, minimum microbubble dosage, burst duration, number of bursts) from which BBB disruption occurred without hemorrhage or edema and to the extent that Evans blue and/or GBCA extravasation occurred. From these experiments, we establish parameters for safe BBB disruption (as reported by H&E staining and immunohistochemistry) in the marmoset at 1.46 MHz. At these parameters, we found that spatial binding of Evans blue staining and GBCA reporting (Fig. 7) were similar, such that the determination of a BBB disruption can be conducted in vivo with MRI-based contrast agents. Taken together, the experiments described here provide an account from which in vivo, minimally invasive substance delivery experiments can be designed around.

As with the rodent brain, marmosets have a lissencephalic cortex, making this species an ideal candidate for tFUS-based BBB disruption, allowing for sonications along the columnar organization of the cortex, unencumbered by cortical folds present in most other primate species. As we show here, however, sonication parameters from rodent species (e.g., rats, mice)^8^ cannot be simply ported for use in the marmoset, nor can those used in for other larger nonhuman primate species such as macaques (with a much thicker skull, and folded cortex)^4^. Indeed, in addition to the parameters driving the transducer, the physical properties of transducers also complicate comparisons (e.g., focal length, frequency). The experiments presented here demonstrate that a 35 mm single element spherically focused 1.46 MHz transducer can be used for cortical disruptions in the marmoset with minimal deleterious effects of the marmoset head morphology (e.g., skull thickness or the presence of temporalis muscles; Fig. 5), particularly when the center of focus is at or near the surface of cortex (Figs. 1–8), allowing for further spatial minimization of cortical disruption. The use of a single-element transducer rather than an array of transducers simplifies the means necessary to conduct tFUS in the marmoset. We made use of a motorized positioning system and automated atlas targeting, but these experiments could also be conducted with a transducer mounted to a stereotactic manipulator arm, further simplifying the equipment needed to use focused ultrasound to sonicate the marmoset brain.

Although it should be noted that microbubble experiments (dosage, clearance time) may be parameter and transducer-dependent^28^, we found that the minimum microbubble dosage (Definity, Lantheus Medical Imaging, Billerica, MA, USA), via the tail- or saphenous-vein was greater than 20 μl/kg when injected as a bolus (Figs. 3 and 9b for % of successful BBB extravasations as a function of dose). To target a smaller distribution of microbubbles (with larger bubbles being more buoyant), we drew from as close to the bottom of the microbubble vial as possible (after activation and slowly inverting the vial) with a 21-gauge needle, and another 21-gauge needle to vent the vial. We also chose to use a 26-gauge catheter to avoid premature destruction of the microbubbles during injection of our saline-diluted microbubble solution. During initial experiments, we found that the use of a spring-loaded extension led to inconsistent BBB perturbation, likely due to premature bursting of the microbubbles. As such, all injections were made directly into the catheter hub. In terms of microbubble clearance time, we found it to be relatively fast (<2 min) in the marmoset, even with a high dose of 200–400 μl/kg—at least to the extent that the circulating microbubble concentration resulted in BBB disruption (Fig. 4). We found that the acoustic emissions indicated cavitation of the microbubbles at 30 s, as indicated by increased broadband signal around the subharmonic. In particular, consistent with previous reports in rats^8^ using a similar transducer and hydrophone hardware, subharmonic broadband noise was evident at 30 and 120 s post microbubble bolus injection (Supplementary Fig. 2); it is worth noting that our hydrophone was sensitized to subharmonic cavitation rather than ultra-harmonic signals, with sensitivity to ~73 kHz orders of magnitude higher than at the harmonic and ultraharmonic frequencies. This likely corresponded to microbubble cavitation, although it did not correspond to opening at 120 s post-microbubble injection. This subharmonic effect was not apparent at 240- or 480-s post-microbubble injection.

Our original intent to determine BBB disruption as a function of burst duration and number of bursts was not to assess damage (visible to the naked eye, Fig. 6), but as shown by the H&E staining (Supplementary Fig. 3), the pressure used for marmoset SK was above of what can be considered safe (Fig. 8). These data, however, are particularly useful for demonstrating the extent of damage that can occur at the extremes of acoustic pressure, burst durations, and at high microbubble dosages (400 μl/kg). We found that these sonications resulted in diffuse tissue damage usually accompanied by microbleeds. Of particular interest, we found that the subdural space presented hemorrhage. This area is particularly sensitive for hemorrhages due to the numerous presence of perforating arteries in a compact space and thus the microbubble concentration may have been higher. Compared to other species, the derated pressures (Fig. 9) are in line with safe BBB opening and also pressure that cause cortical tissue damage, with mechanical indices here ranging from ~0.26 to 1.40^29–37^. It is important to note that although we sonicated multiple locations for the purpose of testing opening parameters, redisruption associated with sterile inflammation can occur^38,39^ when sonicating adjacent locations. Here, we sonicated a grid with a minimum distance of 8 mm, with the exceptions of Marmosets T and G in which an additional 4 sonications occurred along parietal cortex (albeit as shown in Fig. 4, those sonications did not result in BBB opening). As such, the parameters presented here should be considered for single sonications (or sparsely spaced multiple sonications), not repeated proximal sonications as shown in ref. ^38^.

Given the results of the H&E staining of the animals that showed reliably successful disruptions of the BBB (Figs. 2–6), we applied what we found to be safe parameters (derated acoustic pressure = 0.95 MPa, burst duration = 20 ms, burst period = 1000 ms, number of bursts = 60, microbubble dose = 100 μl/kg) to a frontal site (area 8a) in marmoset G to determine the length of time that the BBB remained open, as indexed by permeability to a bolus of GBCA at 2, 4, and 8 h after sonication. The BBB was clearly open at 8 h post-sonication, as indicated by increased intensity and distribution resulting from the GBCA and at the start of the 8-h post-sonication scan (Fig. 7). Although this long-duration disruption may present some risk (e.g., blood-borne bacteria) that the BBB would normally protect against circulating toxins or pathogens in the bloodstream^40^, it is an experimentally advantageous treatment window for injecting substances that may be dangerous if injected as a bolus, rather than slowly infused. The histology and immunohistochemistry in marmoset G supported the previous results that the parameters were safe, such that no readily apparent damage was observed in the H&E staining (Figs. 8 and 10). There is visible microglial activation (Fig. 7) due to tissue perturbation (Iba1), but not tissue damage (DAPI, NeuN, H&E) compared to a contralateral non-sonicated 8a site indicating that these are safe and reliable parameters to open the BBB for an extended period. Microglia in cortical regions showed signs of activation through increased Iba1 expression and changes in cell bodies and processes, without significant changes in cell numbers. FUS-induced BBB disruption has been shown to trigger transient glial activation^38^. Depending on the type and severity of brain injury, activated microglia as well as infiltrating macrophages can exacerbate neuroinflammation and neurodegeneration. It has been demonstrated, however, that microglia activation resolved by 15 days after FUS with no progression to a glial scar, suggesting that FUS does not cause lesion-like microgliosis^41^.

In summary, we demonstrate safe and effective disruption of the BBB in the marmoset with a spatial specificity of ~1 mm radially and 2.5 mm axially (in cortex) using a 1.46 MHz transducer. We were able to reliably perturb the BBB across the dorsal surface of the marmoset cortex with a minimum derated acoustic pressure to be between 0.95–1.16 MPa and a minimum microbubble dosage of 20 μl/kg via tail-vein injection. Further, we found that on average, across the marmoset skull, acoustic pressure at 1.46 MHz was derated by 47%. We demonstrate that these parameters (paired with 60 20 ms bursts, spaced at 1000 ms) led to the BBB being open for greater than 8 h and did not lead to cortical tissue damage, as reported by H&E staining. Consistent with previous reports in other species, we found that acoustic pressure accounted for the most variance among the variables tested and was linearly related to extravasation volume^32,42,43^. Higher acoustic pressures and/or excessive microbubble dosage (>200 μl/kg) led to tissue damage (Fig. 8). The series of experiments presented here establish methods for safely, reproducibly, and focally perturbing the BBB using tFUS in the common marmoset monkey.

## Methods

### Animals

Nine adult marmosets (*Callithrix jacchus*) contributed data to this study (Supplementary Table 1). Animals were anesthetized (induced and maintained) with 2% isoflurane delivered via mask for both the tFUS and in vivo MRI procedures. During the procedures, heart rate, blood oxygenation, respiration, and rectal temperature were monitored. The head was shaved with clippers, then any remaining hair was removed with depilatory cream. A 26-gauge catheter (1/2- or 3/4-inch length) was placed in the lateral tail or saphenous vein for microbubble and contrast agent delivery. Body temperature was maintained with infrared or heated water blankets. Experimental procedure complied with the ethical guidelines for animal testing approved by the University of Pittsburgh Institutional Animal Care and Use Committee.

### Focused ultrasound apparatus

Sonications were performed with the RK-50 Marmoset (FUS Instruments Incorporated, Toronto, ON, Canada), which was developed for use in marmosets in collaboration with FUS Instruments to implement marmoset-specific hardware, including a marmoset stereotaxic device (Model SR-AC; Narishige International Incorporated, Amityville, New York, USA) and to include an MRI-based marmoset atlas for stereotactic targeting^25,26^ (Fig. 1). The system uses an automatic 3-axis positioning system that is configured with reference to stereotactic position on the treatment planning workstation running MORPHEUS software (MORPHEUS framework, FUS Instruments Incorporated, Toronto, ON, Canada). The 3-axis positioning system guided one of two 35 mm spherically focused and calibrated transducers used in this study, either a 515 kHz or 1.46 MHz transducer (FUS Instruments Incorporated, Toronto, ON, Canada) was rigidly mounted to the positioning system to allow for precise control of the sonication location. The sonication parameters—amplitude, number of pulses, repetition period, and number of bursts—were set in the MORPHEUS software package. For each transducer, the voltage-to-pressure values were calibrated from the manufacturer and as described in the following section, we validated the performance of the 1.46 MHz transducer on-site. The number of pluses, repetition period, and number of bursts commanded by the software were generated by an external waveform generator (Siglent SDG 1032X, Siglent Technologies, Solon, Ohio, USA) and sent through a 15 W amplifier to the transducer. A digital oscilloscope was used to monitor cavitation emissions from the hydrophone, which was mounted concentrically with the transducers. As illustrated in Fig. 1a, the transducer was sealed with a 3D printed cover with an o-ring seal and a polyimide film face that held degassed water (via portable water degasser; FUS-DS-50, FUS Instruments Incorporated, Toronto, ON, Canada). The sensor and cover were immersed in a tank holding ~300 ml of degassed water. The polyimide base of the tank was coupled with the head via ultrasound gel.

### Assessment of performance and accuracy

To validate the performance of the FUS system and accurately quantify what parameters correspond to tissue damage or inflammation, we measured the benchtop output of the 1.46 MHz transducer using a capsule hydrophone (HGL-0085, ONDA Corporation, Sunnyvale, CA, USA) and digital oscilloscope (WaveRunner 6051A, Teledyne LeCroy, Chestnut Ridge, NY, USA). Pulse duration (2 ms), burst period (500 ms), and frequency (1.46 MHz) as well as peak negative voltage linearity (between 0.2 and 3.8 MPa) were commanded through the MORPHEUS software and confirmed through hydrophone readings in a degassed water bath with the hydrophone set to the center of focus with a rigid transducer-to-hydrophone bracket.

With the temporal accuracy corroborated, we then tested the spatial accuracy the 1.46 MHz transducer, as mounted in the same 3-axis positioning we used for targeting in the marmosets. To do so, we designed and milled (Roland Modela MDX-50) a custom acrylic plate (polymethyl methacrylate, #8589K922, McMaster-Carr Supply Company, Elmhurst, IL, USA) to be mounted by the ear bar thumb screws of the stereotax, then centered in the same relative position as the marmoset brain (see Supplementary Fig. 1a). Acrylic was chosen for its relatively low melting point of 160 degrees C and relatively high flexural modulus of 490 kip/si, such that it could be milled accurately, mounted in the stereotaxic with minimal flex, while also melting with minimized energy input, minimizing potential damage to the transducer element. The duration of a continuous wave sonication was increased until melting occurred (10 s at 2.2 MPa), then 24 points were sonicated in a 20 × 20 mm grid, with 5 mm spacing between points and the center point (representing 0 mm) defined by a milled point from which the stereotactic coordinates were aligned (see Supplementary Fig. 1b). After melting the grid of points, the acrylic plate was placed in a 200 ml bath potassium iodine at 5 mM (M5005; Sigma-Aldrich Co., MO) and scanned with a small animal CT (Si78; Bruker BioSpin GmbH, Ettlingen, Germany) using a Low Dose 1 mm aluminum filter, 200 × 200 × 200 μm resolution (field of view = 79.6 × 81.1 mm) and a “step and shoot” method (0.6-degree gantry step) and reconstructed using the filtered back projection algorithm. By submerging the plate in iodine, we were better able to see the contours of the melted acrylic, when compared to scanning in open air (Supplementary Fig. 1c–f). To compare the melted acrylic sheet with the target locations, we generated a 3D CAD file with the ideal target locations, converted that file to NIfTI format and registered it to the CT image based on the milled zero point (Supplementary Fig. 1f, d) using FSLeyes (FMRIB Software Library)^44^. Once aligned, the peak of the melted area was determined for each point and spatial deviations (Euclidean distance) from the ideal location were analyzed using MATLAB 2023a (Mathworks) and plotted using built-in functions quiver and imagesc.

#### Acoustic pressure attenuation of the marmoset skull

Using a 1.46 MHz transducer and the hydrophone setup described above, we tested deration of 2.2 MPa sonications through six degassed marmoset skulls and compared those values to free-field measurements in degassed water. Averaged across 6 adult marmoset skull caps (cut in stereotactic plane, starting at the top of the eye ridge, but fully intact dorsal to that), we found that on average the deration was 47% with the shaved scalp, and 44% with just the skull and attached muscle (35–42 points were tested systematically across each skull, depending on skull size, and spaced at 2 mm). As such, we used the average value of 53% to derate our commanded values across the manuscript. Using these same samples, we also compared the attenuation effect of bone without attached muscle (at midline) to that through the thickest portion of the temporalis muscle, where the bone was also thickest (at 10 mm lateral from midline, and 5 mm anterior to the interaural plane). Where the bone and muscle were thickest, there was an additional 2.5% of attenuation. When a detached temporalis muscle was placed between the transducer and hydrophone, no measurable attenuation effect was observed, and thus this effect was due to bone thickness or skull angle alone.

### Stereotactic atlas-based targeting

Sonications were applied transcranially (with skin intact, only hair removed) based on stereotactic position, with *x* = 0, *y* = 0, *z* = 0 mm corresponding to midway between the center of the ear bars, in plane with the bottom of the orbit bars. The marmoset-specific stereotax was rigidly mounted the RK-50 Marmoset baseplate with thumbscrews. Sonication sites were then specified based on the desired location in stereotactic space (Fig. 1b for example). For co-localization with functional and structural atlas landmarks in the marmoset brain, the tFUS positioning software (MORPHEUS framework, FUS Instruments Incorporated, Toronto, ON, Canada) was integrated with the marmosetbrainmapping.org^45^ and marmosetbrainconnectome.org^26^ atlases. Together with the cytoarchitectonic boundaries derived from the Paxinos marmoset brain atlas^24^, the Morpheus software allowed for accurate positioning of the transducer with reference to the adult marmoset brain.

### Sonications

#### Microbubbles

Immediately prior to the sonication (<1 min) microbubbles (Definity, Lantheus Medical Imaging, Billerica, MA, USA), were administered via lateral tail or saphenous vein catheter to aide in BBB disruption. Microbubble solutions were injected directly into the catheter hub; a 26-gauge catheter was chosen to reduce the probability of premature microbubble destruction. The concertation (20–400 μl/kg) of microbubbles varied across experiments (detailed in turn), but all injections were prepared in a stock solution (100 μl microbubbles/860 μl sterile saline) in a 1 ml syringe (weight (kg) × microbubble concertation (ml/kg) × 9.6 = injection volume (ml)) for all experiments. The solution was injected as a bolus and flushed with 200 μl of sterile saline to ensure that the microbubbles cleared the volume of the catheter hub.

#### Evans blue and MRI contrast agent injections

Evans blue stain and a gadolinium-based MRI contrast agent (GBCA) were used to verify BBB disruption. All agents were injected immediately after the last sonication as a bolus (albeit the injection timing varied for the BBB disruption duration and clearance experiments, detailed below). Evans blue (E2129; Sigma-Aldrich Co., MO) was injected intravenously as a bolus in all animals at a dose of 2 μl/g in 2% solution, prepared in sterile water. Gadolinium (Gadavist^TM^, gadobutrol; Bayer Healthcare Pharmaceuticals, Leverkusen, Germany) was prepared in 200 μl of sterile saline and injected at a dose of 100 μl/kg in all marmosets except Marmosets G (200 μl/kg) and SP (600 μl/kg).

#### BBB disruption as a function of center frequency

Marmoset B received two sonications in parietal cortices to determine the relative difference in BBB disruption size as a function of transducer center frequency (Fig. 1c). With previous reports of reliable BBB disruption in rats (who have a similar skull thickness and brain size to marmosets) at ~500 kHz and its harmonic ~1.5 MHz^29^ we tested both a 515 kHz and 1.46 MHz transducer, but as we go on to show, the size of opening from 1.46 MHz was more suitable for the experiments described in this study and thus all other sonications were performed at 1.46 MHz. The only sonication performed at 515 kHz was in left parietal cortex of Marmoset B (1 MPa commanded, derated ~0.5 MPa), 30 ms burst duration, 1000 ms burst period, 90 bursts, 400 μl/kg microbubble dose). Right parietal cortex in the same animal was sonicated with a 1.46 MHz transducer (1.70 MPa (derated), 30 ms burst duration, 1000 ms burst period, 90 bursts, 400 μl/kg microbubble dose). BBB disruption was reported by Evans blue staining ex vivo.

#### BBB disruption as a function of acoustic pressure

For this experiment, marmosets SG, NE, and T each received five or eight cortical sonications (area 8a, 4ab, MIP, and V2) with a 1.46 MHz transducer. Figure 2 shows the sonication locations and accompanying derated acoustic pressures. For Marmoset SG, the acoustic pressure was modulated with the following parameters remaining constant: burst duration = 30 ms, burst period = 1000 ms, number of bursts = 90, microbubble dose = 400 μl/kg. Based on the information gained from Marmoset SG, Marmosets NE and T were sonicated at lower acoustic pressures with the following parameters remaining constant: burst duration = 20 ms, burst period = 1000 ms, number of bursts = 60, microbubble dose = 200 μl/kg. The purpose of this experiment was threefold: to (1) determine the minimum acoustic pressure to open the BBB in a marmoset at 1.46 MHz, (2) determine the size/shape of BBB disruption as a function of acoustic pressure, and (3) determine the pressure at which damage starts to occur. For all three animals, BBB disruption was reported by in vivo GBCA-enhanced MRI and Evans blue staining ex vivo. Tissue damage was assessed using H&E staining (detailed below).

#### Minimum microbubble dosage to open the BBB

Marmosets NE and T were used to determine the minimum microbubble dosage necessary to open the BBB. Each animal was sonicated four times (area 8a, 4ab, MIP, and V2), with differing microbubble dosages from 0 to 200 μl/kg (Fig. 3 shows dosages at specific locations), with the following parameters remaining constant: derated acoustic pressure = 1.17 MPa, burst duration = 20 ms, burst period = 1000 ms, number of bursts = 60. Ten minutes elapsed between sonications and microbubble injections to reduce the confounding effects of circulating microbubbles. Note that Marmosets NE and T participated in both the acoustic pressure (above) and microbubble dosing experiment (albeit separate sonications sites, except for left 8aD, which informed both experiments). For both animals, BBB disruption was reported by in vivo GBCA-enhanced MRI and ex vivo Evans blue staining. Tissue damage was assessed using H&E staining.

#### Microbubble clearance

Marmosets T and M received four sonications (parietal areas PFG right, PE right, PE left, and PFG left; albeit slightly different loci, see Fig. 4 for monkey-wise locations) to determine the ability to open the BBB across multiple sites after a single bolus injection of microbubbles. Figure 4 shows separate sonication locations beginning at 30, 60, 90, 120, 240, and 480 s after a bolus injection of 200 μl/kg of microbubbles. For all sites, a 1.46 MHz transducer was used following parameters remaining constant: derated acoustic pressure = 1.17 MPa, burst duration = 20 ms, burst period = 1000 ms, number of bursts = 60. BBB disruption was reported by in vivo GBCA-enhanced MRI (note that the MRI sequence varied for Marmosets T and M, but both clearly showed T1-weighted GBCA contrast) and ex vivo Evans blue staining. Tissue damage was assessed using H&E staining. Frequency spectra from acoustic emissions were generated using a fast Fourier transform and averaged across pulses (Supplementary Fig. 2).

#### BBB disruption as a function of skull angle

Marmoset SP received two sonications (left: 6DC; right: area 6VA). Unlike the previous experiments, the sonications were not delivered symmetrically—the right hemisphere sonications were translated lateral from the left hemisphere sonications to additionally vary skull angle along the medial-lateral axis (Fig. 5 for locations). Both sonications used the following parameters with a 1.46 MHz transducer: derated acoustic pressure = 1.70 MPa, burst duration = 30 ms, burst period = 1000 ms, number of bursts = 90, microbubble dosage = 400 μl/kg. A high-resolution computed tomography (CT) image was acquired to calculate skull angle and coregistered to template space^25^. BBB disruption size was calculated with the FIJI software package^46^ based on the Evans blue staining microscopy images (Fig. 5e).

#### BBB disruption as a function of burst duration and number of bursts

Marmosets SK received 8 total sonications (area 8a, 4ab, MIP, and V2 bilaterally). In the left hemisphere, duty cycle was varied from 5 to 20 ms (Fig. 6 for duty cycle by location), with the following parameters held constant with a 1.46 MHz transducer: derated acoustic pressure = 1.70 MPa, burst period = 1000 ms, number of bursts = 60, microbubble dosage = 400 μl/kg. In the right hemisphere, the number of bursts was varied from 5 to 40 bursts (Fig. 6 for number of bursts by location), with the following parameters held constant with a 1.46 MHz transducer: derated acoustic pressure = 1.70 MPa, burst duration = 20 ms, burst period = 1000 ms, microbubble dosage = 400 μl/kg. Marmoset M received 6 total sonications with the same 1.46 MHz transducer, but with reduced parameters and microbubble dose than marmoset SK: derated acoustic pressure = 1.17 MPa, burst period = 1000 ms, microbubble dosage = 200 μl/kg. In the left hemisphere (Fig. 6 for locations), burst duration was varied from 5 to 20 ms; in the right hemisphere, the number of bursts was varied from 5 to 20 bursts (note, again, that the MRI sequence varied for Marmosets SK and M, but both clearly showed T1-weighted GBCA contrast).

#### BBB disruption duration

Marmoset G received a single sonication (area 8a, right hemisphere) with a 1.46 MHz transducer and the following parameters: derated acoustic pressure = 0.95 MPa, burst duration = 20 ms, burst period = 1000 ms, number of bursts = 60, microbubble dosage = 100 μl/kg. BBB disruption was reported by in vivo GBCA-enhanced MRI. To determine the duration that the BBB remained open, Marmoset G was administered three bolus injections of a GBCA at 2-, 5-, and 8-h post-sonication. MPRAGE anatomical images (sequence detailed below) were collected hourly to determine the changes in the size and intensity of the GBCA passing the BBB into parenchyma. BBB disruption was also reported via Evans blue, injected immediately after the last sonication. Tissue damage was assessed using H&E staining, accompanied by IBa1, NeuN, and DAPI. Marmoset M also received a single sonication in area 8a (derated acoustic pressure = 1.17 MPa, burst duration = 20 ms, burst period = 1000 ms, number of bursts = 60, microbubble dosage = 200 μl/kg), but the GBCA was administered at 2 h, then a much longer period of 2 weeks after sonication. Given the survival time necessary for the experiment (2 weeks), Marmoset M did not receive Evans blue.

### Magnetic resonance imaging

#### In vivo MRI, CT

All neuroimaging (MRI and CT) took place at the University of Pittsburgh Brain Institute. For MRI, a 9.4 T 30 cm horizontal bore scanner (Bruker BioSpin Corp, Billerica, MA) was used, equipped with a Bruker BioSpec Avance Neo console and the software package Paravision-360 (version 3.2; Bruker BioSpin Corp, Billerica, MA), and a custom high performance 17 cm gradient coil (Resonance Research Inc, Billerica, MA) performing at 450 mT/m gradient strength. To detect BBB disruption in vivo, MRI was acquired on Marmosets B, SG, NE, T, M, SK, SP and G in concert with an intravenous contrast agent (gadolinium) that was injected after the sonications (within 1 min of last sonications, except Marmoset E to determine BBB disruption duration) and before the MRI (MRI started within 20 min of transferring the animal from the FUS including scanner preparations involving localization and magnetic field shimming). Radiofrequency transmission was accomplished with a custom 135 mm inner diameter coil and a custom in house 8-channel phased-array marmoset-specific coil was used for radiofrequency receiving. Marmosets were imaged in the sphinx position, with a custom 3D printed helmet for head fixation and anesthesia mask for inhalant isoflurane delivery. A T1-weighted fast low angle shot (FLASH) sequence was employed to detect the resultant shortening of T1 relaxation times from the contrast agents entering the parenchyma via the BBB disruption. Three scans (later averaged) were acquired for each animal with the following parameters: TR = 25 ms, TE = 8 ms, field of view = 35 × 35 × 26 mm, matrix size = 117 × 117 × 87, voxel size = 0.299 × 0.299 × 0.299 mm, bandwidth = 200 kHz, flip angle = 25 degrees, total scan time = 9 min, 2 s. For marmosets G & M, a magnetization prepared—rapid gradient echo (MPRAGE) sequence was used in lieu of the FLASH sequence because of the longitudinally-additive effects of systemic GBCA on the dynamic signal contrast. With the MPRAGE sequence, this additive effect is reduced with the additional inversion pulse. The MPRAGE sequence was acquired with the following parameters: TR = 6000 ms, TE = 3.42 ms, field of view = 42 × 35 × 25 mm, matrix size = 168 × 140 × 100, voxel size = 0.250 × 0.250 × 0.250 mm, bandwidth = 50 kHz, flip angle = 14 degrees, total scan time = 20 min, 6 s.

To accurately quantify skull angle relative to the ultrasonic transducer, CT was acquired on Marmoset SP at the University of Pittsburgh Brain Institute on a small animal CT (Si78; Bruker BioSpin GmbH, Ettlingen, Germany) equipped with the software package Paravision-360 (version 3.1; Bruker BioSpin Corp, Billerica, MA). Using a Low Dose 1 mm aluminum filter, a 200 × 200 × 200 μm (field of view = 79.6 × 81.1 mm) CT was acquired using a “step and shoot” method (0.6-degree gantry step) and reconstructed using the filtered back projection algorithm. The MRI and sliced microscopy images were then aligned using the 2D to 3D registration provided in DSIstudio^47^.

#### Quantification of extravasated volume across the BBB

Quantification of BBB “opening size” was calculated with the post-sonication MRIs from marmoset SG, SK, NE, T, G, and M by measuring the volume of extravasated GBCA at the center of the respective sonication point in AFNI’s Draw Dataset Plugin^48^. The resultant volumes were calculated using AFNI’s 3dROIstats and loaded into MATLAB 2023a (Mathworks) for further statistical analysis across animals and parameters. We performed linear regression (“fitlm” and “stepwiselm” functions, Statistics and Machine Learning Toolbox) with input variables of derated acoustic pressure, microbubble dose, number of bursts, burst period, and animal weight. Calculated volume of extravasated GBCA was the dependent variable. Three total regression analyses were conducted: (1) linear regression of the aforementioned variables, with extravasation volume as the dependent variable, (2) the same, but as a stepwise regression, and (3) the same independent variables in a linear regression, but with extravasation volume binarized (i.e., 1 = gadolinium contrast detected, 0 = no GBCA contrast detected).

### Histology and immunohistochemistry

After in vivo MRI acquisition (and 6–8 h after sonications), all nine marmosets were euthanized with pentobarbital sodium and phenytoin sodium solution (100 mg/kg) for histological examination. Transcardial perfusion was performed with 4% paraformaldehyde. The brains were removed, postfixed, and cryoprotected in 30% sucrose for 3–5 days. Marmoset brains were sectioned coronally at 30 μm using a cryostat (Leica CM1950, Deer Park, IL, USA) and stored in cryoprotectant solution with 15% glycerol and 15% ethylene glycol at −20 °C until further use. For Evans blue fluorescence, sections were mounted onto Superfrost slides (Fisher Scientific) and visualized under an AxioImager M2 epifluorescence microscope (Car Zeiss, White Plains, NY, USA). For Hematoxylin and Eosin (H&E) staining, sections were mounted onto a Superfrost slide and stained with an H&E stain kit (#3502 Vector Laboratories, Newark, CA, USA) by following procedures suggested by the manufacturer. Images were captured using an AxioImager M2 microscope (Carl Zeiss). For immunofluorescence staining, floating sections were permeabilized in blocking buffer (2% donkey serum and 0.2% Triton X-100 in PBS) at room temperature for 1 h with gentle shaking, followed by overnight incubation with primary antibodies, Iba1 (Ionized calcium-binding adapter molecule 1—1:500; #019-19741 Wako Chemicals) and NeuN (Fox-3—1:500; #MAB377 MilliporeSigma) at 4 °C. After PBS wash, sections incubated with fluorescent secondary antibodies, Alexa Fluor 488-conjugated donkey anti-rabbit IgG (Invitrogen) and Alexa Fluor 647-conjugated donkey anti-mouse IgG (Invitrogen). Sections were counterstained with DAPI (4’,6-Diamidine-2’-phenylindole dihydrochloride—Invitrogen) for staining nuclei. Images were acquired using a LSM900 confocal microscope (Carl Zeiss) using ×10 objective at 1024 × 1024 pixel resolution with z-step size of 1 μm thickness.

As a more sensitive assessment of damage^38^, additional TUNEL staining (PK101; FD NeuroTechnologies, Columbia, MD USA) was performed on Marmoset G and SK.  CD68 (MCA1957; Bio-Rad, Hercules, CA USA) and CD206 (ab64693; abcam, Waltham, MA, USA) were also used to stain slices from Marmoset G as a marker of activated phagocytic microglia. Immunostaining against GFAP (C9205; Sigma-Aldrich, St. Louis, MO USA) was used to identified astrocytes. Lectin (DL-1174-1; Vector Laboratories, Newark, CA USA) and the nuclear marker DAPI (62248; Thermo Fisher Scientific) were used to visualize blood vessels.

#### Statistics and reproducibility

Parameters (acoustic pressure, microbubble dosage, number of bursts, burst period) were varied and tested across multiple animals of different ages, weights, and sex (Supplementary Table 1). BBB opening volume was quantified using GBCA in vivo MRI using AFNI’s Draw Dataset Plugin and 3dROIStats. Statistics were performed using a combination of in-house code and built-in code from MATLAB’s Statistics and Machine Learning Toolbox, specifically “fitlm” and “stepwiselm” functions. Linear regression was performed across animals and used all varied parameters as well as the animal’s weight.

### Reporting summary

Further information on research design is available in the Nature Portfolio Reporting Summary linked to this article.

## Supplementary information

Supplementary Material

Description of Additional Supplementary Files

Supplementary Data 1

Reporting Summary

## Data Availability

Data available upon reasonable request from the authors. Source data for Fig. 9 can be found in Supplementary Data 1.

## Code availability

Code available upon reasonable request from the authors.

## References

[1] Pardridge WM (2007). Drug targeting to the brain. Pharm. Res.

[2] Hynynen K, McDannold N, Vykhodtseva N, Jolesz FA (2001). Noninvasive MR imaging–guided focal opening of the blood-brain barrier in rabbits. Radiology.

[3] McDannold N, Vykhodtseva N, Hynynen K (2006). Targeted disruption of the blood-brain barrier with focused ultrasound: association with cavitation activity. Phys. Med. Biol..

[4] McDannold N, Arvanitis CD, Vykhodtseva N, Livingstone MS (2012). Temporary disruption of the blood-brain barrier by use of ultrasound and microbubbles: safety and efficacy evaluation in rhesus macaques. Cancer Res..

[5] Pacia CP (2020). Feasibility and safety of focused ultrasound-enabled liquid biopsy in the brain of a porcine model. Sci. Rep..

[6] Choi JJ, Pernot M, Small SA, Konofagou EE (2007). Noninvasive, transcranial and localized opening of the blood-brain barrier using focused ultrasound in mice. Ultrasound Med. Biol..

[7] Karakatsani MEM, Samiotaki GM, Downs ME, Ferrera VP, Konofagou EE (2017). Targeting effects on the volume of the focused ultrasound-induced blood-brain barrier opening in nonhuman primates in vivo. IEEE Trans. Ultrason. Ferroelectr. Freq. Control.

[8] Lapin NA, Gill K, Shah BR, Chopra R (2020). Consistent opening of the blood brain barrier using focused ultrasound with constant intravenous infusion of microbubble agent. Sci. Rep..

[9] Kobus T, Vykhodtseva N, Pilatou M, Zhang Y, McDannold N (2016). Safety validation of repeated blood-brain barrier disruption using focused ultrasound. Ultrasound Med. Biol..

[10] Ozdas MS (2020). Non-invasive molecularly-specific millimeter-resolution manipulation of brain circuits by ultrasound-mediated aggregation and uncaging of drug carriers. Nat. Commun..

[11] Trinh D (2022). Microbubble drug conjugate and focused ultrasound blood brain barrier delivery of AAV-2 SIRT-3. Drug Deliv..

[12] Noroozian Z (2019). MRI-guided focused ultrasound for targeted delivery of rAAV to the brain. Methods Mol. Biol..

[13] Meng Y, Hynynen K, Lipsman N (2021). Applications of focused ultrasound in the brain: from thermoablation to drug delivery. Nat. Rev. Neurol..

[14] Schaeffer DJ (2020). Divergence of rodent and primate medial frontal cortex functional connectivity. Proc. Natl Acad. Sci. USA.

[15] Schaeffer DJ (2019). Intrinsic functional clustering of anterior cingulate cortex in the common marmoset. Neuroimage.

[16] Schaeffer DJ, Gilbert KM, Gati JS, Menon RS, Everling S (2019). Intrinsic functional boundaries of lateral frontal cortex in the common marmoset monkey. J. Neurosci..

[17] Schaeffer, D. J. et al. Face selective patches in marmoset frontal cortex. *Nat. Commun.* **11**, 4856 (2020). 10.1038/s41467-020-18692-2 PMC7519082 32978385

[18] Sheikov N, McDannold N, Vykhodtseva N, Jolesz F, Hynynen K (2004). Cellular mechanisms of the blood-brain barrier opening induced by ultrasound in presence of microbubbles. Ultrasound Med. Biol..

[19] Chowdhury SM, Abou-Elkacem L, Lee T, Dahl J, Lutz AM (2020). Ultrasound and microbubble mediated therapeutic delivery: underlying mechanisms and future outlook. J. Control Release.

[20] Ohl CD (2006). Sonoporation from jetting cavitation bubbles. Biophys. J..

[21] Abrahao A (2019). First-in-human trial of blood-brain barrier opening in amyotrophic lateral sclerosis using MR-guided focused ultrasound. Nat. Commun..

[22] Wang S (2017). Non-invasive, focused ultrasound-facilitated gene delivery for optogenetics. Sci. Rep..

[23] Ogawa K (2022). Focused ultrasound/microbubbles-assisted BBB opening enhances LNP-mediated mRNA delivery to brain. J. Control Release.

[24] Paxinos, G., Watson, C., Petrides, M., Rosa, M. & Tokuno, H. *The Marmoset Brain in Stereotaxic Coordinates* (Academic Press, 2012).

[25] Liu C (2021). Marmoset brain mapping V3: population multi-modal standard volumetric and surface-based templates. Neuroimage.

[26] Schaeffer DJ (2022). An open access resource for functional brain connectivity from fully awake marmosets. Neuroimage.

[27] O’Reilly MA, Muller A, Hynynen K (2011). Ultrasound insertion loss of rat parietal bone appears to be proportional to animal mass at submegahertz frequencies. Ultrasound Med. Biol..

[28] Ferrara K, Pollard R, Borden M (2007). Ultrasound microbubble contrast agents: fundamentals and application to gene and drug delivery. Annu. Rev. Biomed. Eng..

[29] Shin J (2018). Focused ultrasound-mediated noninvasive blood-brain barrier modulation: preclinical examination of efficacy and safety in various sonication parameters. Neurosurg. Focus.

[30] McDannold N, Vykhodtseva N, Hynynen K (2008). Effects of acoustic parameters and ultrasound contrast agent dose on focused-ultrasound induced blood-brain barrier disruption. Ultrasound Med. Biol..

[31] Singh A (2022). Guiding and monitoring focused ultrasound mediated blood–brain barrier opening in rats using power Doppler imaging and passive acoustic mapping. Sci. Rep..

[32] Chen H, Konofagou EE (2014). The size of blood-brain barrier opening induced by focused ultrasound is dictated by the acoustic pressure. J. Cereb. Blood Flow. Metab..

[33] Bing KF, Howles GP, Qi Y, Palmeri ML, Nightingale KR (2009). Blood-brain barrier (BBB) disruption using a diagnostic ultrasound scanner and Definity® in mice. Ultrasound Med. Biol..

[34] Lapin, N. A., Gill, K., Shah, B. R. & Chopra, R. Consistent opening of the blood brain barrier using focused ultrasound with constant intravenous infusion of microbubble agent. *Sci. Rep.* **10**, 16546 (2020). 10.1038/s41598-020-73312-9 PMC7538995 33024157

[35] Hu Z, Chen S, Yang Y, Gong Y, Chen H (2022). An affordable and easy-to-use focused ultrasound device for noninvasive and high precision drug delivery to the mouse brain. IEEE Trans. Biomed. Eng..

[36] McDannold N, Vykhodtseva N, Hynynen K (2008). Blood-brain barrier disruption induced by focused ultrasound and circulating preformed microbubbles appears to be characterized by the mechanical index. Ultrasound Med. Biol..

[37] Chu PC (2016). Focused ultrasound-induced blood-brain barrier opening: association with mechanical index and cavitation index analyzed by dynamic contrast-enhanced magnetic-resonance imaging. Sci. Rep..

[38] Kovacs ZI (2017). Disrupting the blood-brain barrier by focused ultrasound induces sterile inflammation. Proc. Natl Acad. Sci. USA.

[39] Kovacs, Z. I., Burks, S. R. & Frank, J. A. Focused ultrasound with microbubbles induces sterile inflammatory response proportional to the blood brain barrier opening: attention to experimental conditions. *Theranostics* **8**, 2245–2248 (2018). 10.7150/thno.24181 PMC5928885 29722362

[40] Daneman R, Prat A (2015). The blood-brain barrier. Cold Spring Harb. Perspect. Biol..

[41] Jordão JF (2013). Amyloid-β plaque reduction, endogenous antibody delivery and glial activation by brain-targeted, transcranial focused ultrasound. Exp. Neurol..

[42] Lin KJ (2009). Quantitative micro-SPECT/CT for detecting focused ultrasound-induced blood-brain barrier opening in the rat. Nucl. Med. Biol..

[43] Gaur P (2020). Histologic safety of transcranial focused ultrasound neuromodulation and magnetic resonance acoustic radiation force imaging in rhesus macaques and sheep. Brain Stimul..

[44] Smith SM (2004). Advances in functional and structural MR image analysis and implementation as FSL. Neuroimage.

[45] Liu C (2020). A resource for the detailed 3D mapping of white matter pathways in the marmoset brain. Nat. Neurosci..

[46] Schindelin J (2012). Fiji: an open-source platform for biological-image analysis. Nat. Methods.

[47] Yeh FC, Verstynen TD, Wang Y, Fernández-Miranda JC, Tseng WYI (2013). Deterministic diffusion fiber tracking improved by quantitative anisotropy. PLoS ONE.

[48] Cox RW (1996). AFNI: software for analysis and visualization of functional magnetic resonance neuroimages. Comput. Biomed. Res..

