# Supplementary material for: Noninvasive disruption of the blood-brain barrier in the marmoset monkey
Source: Commun Biol. 2023 Aug 2;6:806. doi: 10.1038/s42003-023-05185-3 (PMC10397190; doi:10.1038/s42003-023-05185-3)
Supplement: Supplementary file 1 — Supplementary Material [file 42003_2023_5185_MOESM1_ESM.pdf]

Supplementary Table 1. Summary of animal age, weight, and sex.

**Common marmoset (*Callithrix jacchus*)**	**Age (months)**	**Weight (grams)**	**Sex**
Marmoset B	46	205	F
Marmoset SP	32	195	F
Marmoset SG	24	135	F
Marmoset SK	20	170	F
Marmoset NE	29	175	M
Marmoset T	19	145	F
Marmoset E	18	165	M
Marmoset G	40	215	F
Marmoset M	97	235	M

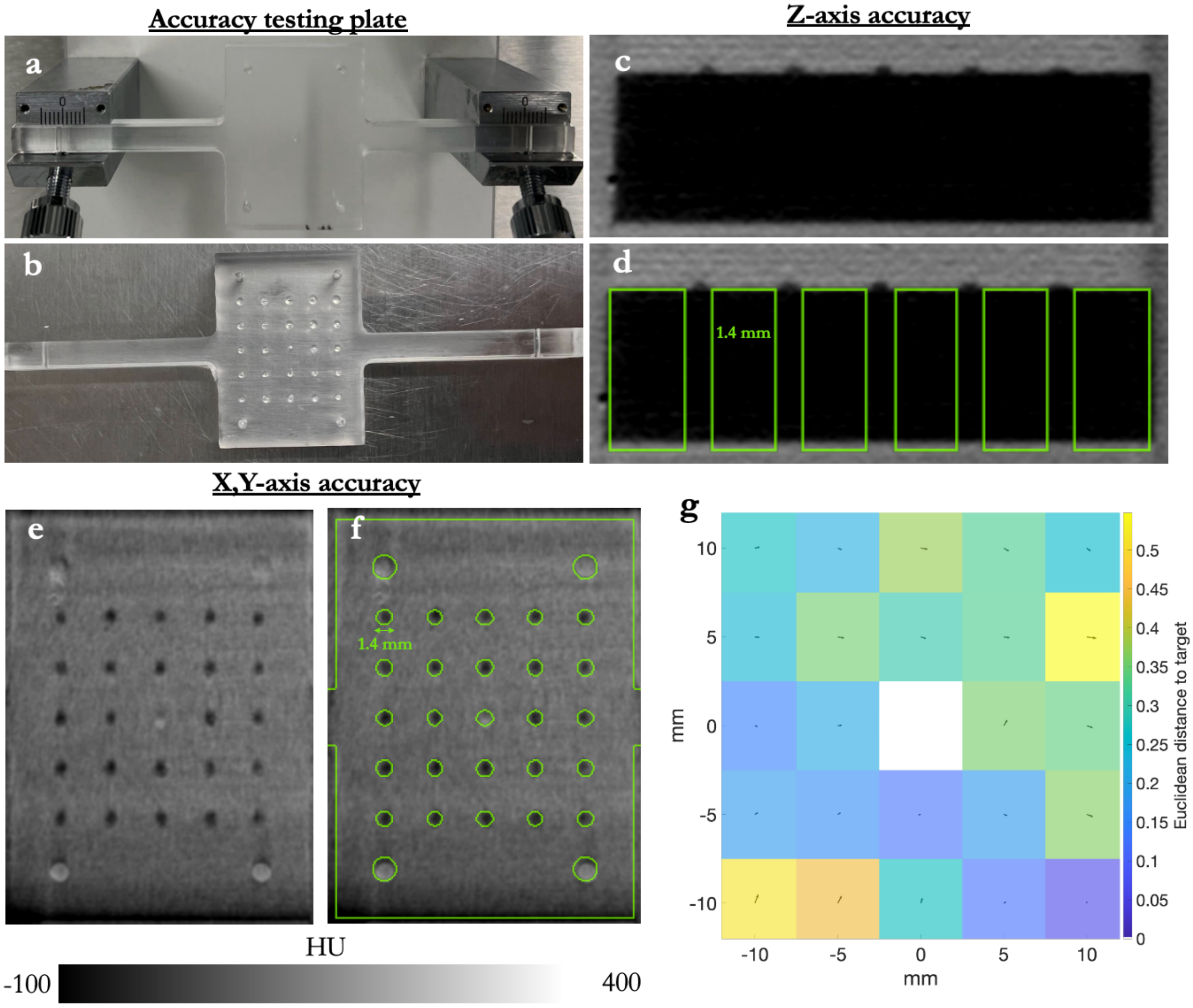

Supplementary Figure 1. Testing spatial accuracy via melting acrylic plate. **a** Acrylic test plate apparatus used for testing spatial accuracy. **b** The same plate, but after 24 melted sonication points. **c** CT of the test plane in the Z plane, with the height of the melted point indicating depth accuracy. **d** Target locations for spatial accuracy test. **e** Same CT image as c but in the X-Y plane **f** Target locations from a CAD file registered to CT image **g** Quantification of accuracy – the Euclidean distance between the melted point and target – with each cell representing a melted point across space, with warmer colors indicating greater accuracy error and arrows indicating the magnitude and direction of the error.

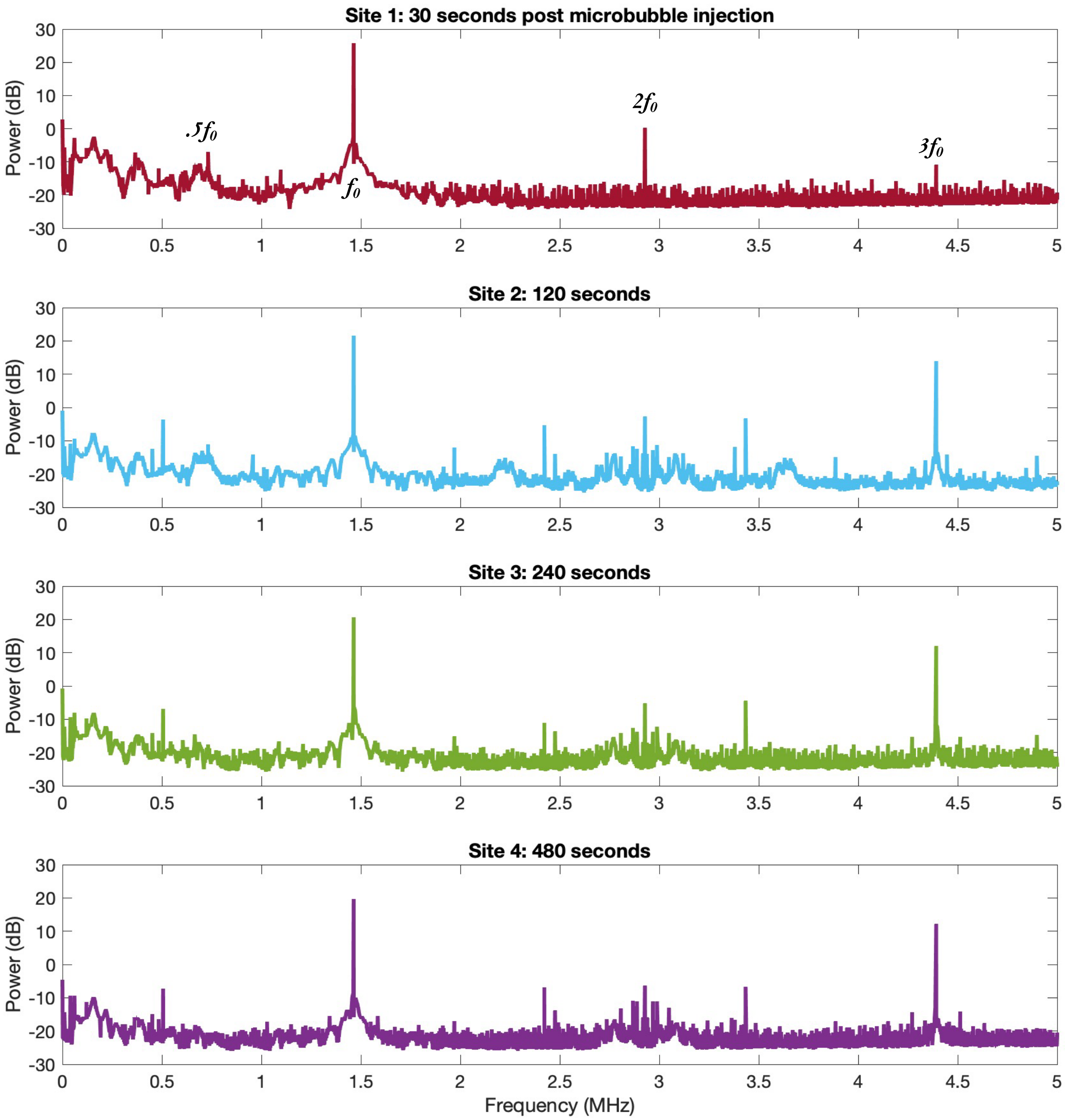

Supplementary Figure 2. Frequency spectra from sonications in marmoset T (sites shown in Figure 4) following a single bolus microbubble injection of 200 µl/kg.

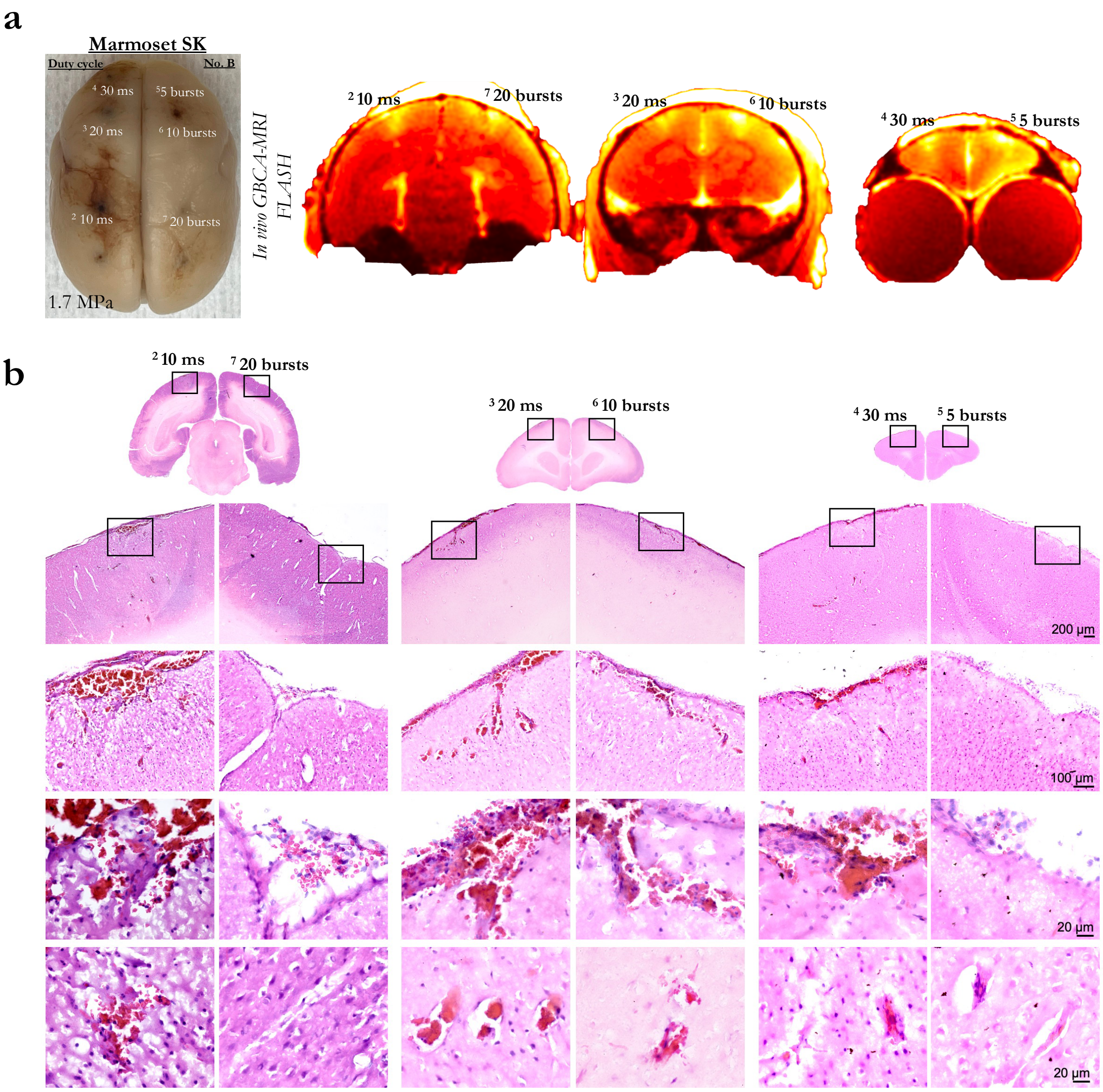

Supplementary Figure 3. Tissue caused by high acoustic pressure and microbubble dose. **a** Images derived from Figure 6 for reference for the sonication locations. **b** H & E staining at the sonicated locations, marked by the black boxes and zoomed to 200, 100, 20 µm.
