# Supplementary material for: Noninvasive disruption of the blood-brain barrier in the marmoset monkey
Source: Commun Biol. 2023 Aug 2;6:806. doi: 10.1038/s42003-023-05185-3 (PMC10397190; doi:10.1038/s42003-023-05185-3)
Supplement: Supplementary file 2 — Description of Additional Supplementary Files [file 42003_2023_5185_MOESM2_ESM.pdf]

Description of Additional Supplementary Files

File name: Supplementary Data 1

Description: The source data behind the plots of Figure 9.
