# Supplementary material for: Noninvasive disruption of the blood-brain barrier in the marmoset monkey
Source: Commun Biol. 2023 Aug 2;6:806. doi: 10.1038/s42003-023-05185-3 (PMC10397190; doi:10.1038/s42003-023-05185-3)
Supplement: Supplementary file 4 — Reporting Summary [file 42003_2023_5185_MOESM4_ESM.pdf]

nature portfolio

Corresponding author(s): David Schaeffer

Last updated by author(s): Jul 20, 2023

Reporting Summary

Nature Portfolio wishes to improve the reproducibility of the work that we publish. This form provides structure for consistency and transparency in reporting. For further information on Nature Portfolio policies, see our Editorial Policies and the Editorial Policy Checklist.

Statistics

For all statistical analyses, confirm that the following items are present in the figure legend, table legend, main text, or Methods section.

n/a	Confirmed	
☐	☒	The exact sample size (*n*) for each experimental group/condition, given as a discrete number and unit of measurement
☐	☒	A statement on whether measurements were taken from distinct samples or whether the same sample was measured repeatedly
☐	☒	The statistical test(s) used AND whether they are one- or two-sided<br>*Only common tests should be described solely by name; describe more complex techniques in the Methods section.*
☐	☒	A description of all covariates tested
☒	☐	A description of any assumptions or corrections, such as tests of normality and adjustment for multiple comparisons
☐	☒	A full description of the statistical parameters including central tendency (e.g. means) or other basic estimates (e.g. regression coefficient) AND variation (e.g. standard deviation) or associated estimates of uncertainty (e.g. confidence intervals)
☐	☒	For null hypothesis testing, the test statistic (e.g. *F*, *t*, *r*) with confidence intervals, effect sizes, degrees of freedom and *P* value noted<br>*Give P values as exact values whenever suitable.*
☒	☐	For Bayesian analysis, information on the choice of priors and Markov chain Monte Carlo settings
☒	☐	For hierarchical and complex designs, identification of the appropriate level for tests and full reporting of outcomes
☒	☐	Estimates of effect sizes (e.g. Cohen's *d*, Pearson's *r*), indicating how they were calculated

Our web collection on statistics for biologists contains articles on many of the points above.

Software and code

Policy information about availability of computer code

Data collection	Paravision 360, Version 3.3.
Data analysis	FMRIB Software Library v6.0<br>AFNI Version AFNI_20.0.19 'Galba'<br>ANTs Version 5<br>Matlab 2023a

For manuscripts utilizing custom algorithms or software that are central to the research but not yet described in published literature, software must be made available to editors and reviewers. We strongly encourage code deposition in a community repository (e.g. GitHub). See the Nature Portfolio guidelines for submitting code & software for further information.

Data

Policy information about availability of data

All manuscripts must include a data availability statement. This statement should provide the following information, where applicable:

- Accession codes, unique identifiers, or web links for publicly available datasets
- A description of any restrictions on data availability
- For clinical datasets or third party data, please ensure that the statement adheres to our policy

Data available on request from the authors.

Research involving human participants, their data, or biological material

Policy information about studies with human participants or human data. See also policy information about sex, gender (identity/presentation), and sexual orientation and race, ethnicity and racism.

Reporting on sex and gender	*Use the terms sex (biological attribute) and gender (shaped by social and cultural circumstances) carefully in order to avoid confusing both terms. Indicate if findings apply to only one sex or gender; describe whether sex and gender were considered in study design; whether sex and/or gender was determined based on self-reporting or assigned and methods used. Provide in the source data disaggregated sex and gender data, where this information has been collected, and if consent has been obtained for sharing of individual-level data; provide overall numbers in this Reporting Summary. Please state if this information has not been collected. Report sex- and gender-based analyses where performed, justify reasons for lack of sex- and gender-based analysis.*
Reporting on race, ethnicity, or other socially relevant groupings	*Please specify the socially constructed or socially relevant categorization variable(s) used in your manuscript and explain why they were used. Please note that such variables should not be used as proxies for other socially constructed/relevant variables (for example, race or ethnicity should not be used as a proxy for socioeconomic status). Provide clear definitions of the relevant terms used, how they were provided (by the participants/respondents, the researchers, or third parties), and the method(s) used to classify people into the different categories (e.g. self-report, census or administrative data, social media data, etc.) Please provide details about how you controlled for confounding variables in your analyses.*
Population characteristics	*Describe the covariate-relevant population characteristics of the human research participants (e.g. age, genotypic information, past and current diagnosis and treatment categories). If you filled out the behavioural & social sciences study design questions and have nothing to add here, write "See above."*
Recruitment	*Describe how participants were recruited. Outline any potential self-selection bias or other biases that may be present and how these are likely to impact results.*
Ethics oversight	*Identify the organization(s) that approved the study protocol.*

Note that full information on the approval of the study protocol must also be provided in the manuscript.

Field-specific reporting

Please select the one below that is the best fit for your research. If you are not sure, read the appropriate sections before making your selection.

☒ Life sciences ☐ Behavioural & social sciences ☐ Ecological, evolutionary & environmental sciences

For a reference copy of the document with all sections, see nature.com/documents/nr-reporting-summary-flat.pdf

Life sciences study design

All studies must disclose on these points even when the disclosure is negative.

Sample size	Sample size was determined by repeatability BBB openings at the individual animal level.
Data exclusions	All acquired data were included.
Replication	Group results were repeatable at the individual animal level
Randomization	Selection not randomized; available animals used.
Blinding	Experimenters were not blinded during the experiment, but identifiers were randomly assigned for analysis

Reporting for specific materials, systems and methods

April 2023

We require information from authors about some types of materials, experimental systems and methods used in many studies. Here, indicate whether each material, system or method listed is relevant to your study. If you are not sure if a list item applies to your research, read the appropriate section before selecting a response.

Materials & experimental systems

n/a	Involved in the study
☐	☒ Antibodies
☒	☐ Eukaryotic cell lines
☒	☐ Palaeontology and archaeology
☐	☒ Animals and other organisms
☒	☐ Clinical data
☒	☐ Dual use research of concern
☒	☐ Plants

Methods

n/a	Involved in the study
☒	☐ ChIP-seq
☒	☐ Flow cytometry
☐	☒ MRI-based neuroimaging

Antibodies

Antibodies used

For immunofluorescence staining, floating sections were permeabilized in blocking buffer (2 % donkey serum and 0.2 % Triton X-100 in PBS) at room temperature for 1 h with gentle shaking, followed by overnight incubation with primary antibodies, Iba1 (Ionized calcium binding adaptor molecule 1 - 1:500; #019-19741 Wako Chemicals) and NeuN (Fox-3 - 1:500; #MAB377 MilliporeSigma) at 4 ⍰ C. After PBS wash, sections incubated with fluorescent secondary antibodies, Alexa Fluor 488-conjugated donkey anti-rabbit IgG (Invitrogen) and Alexa Fluor 647-conjugated donkey anti-mouse IgG (Invitrogen). Sections were counterstained with DAPI (4',6-Diamidine-2'-phenylindole dihydrochloride - Invitrogen) for staining nuclei.

Validation

Validation as per manufacturers' website.

Animals and other research organisms

Policy information about studies involving animals; ARRIVE guidelines recommended for reporting animal research, and Sex and Gender in Research

Laboratory animals

Nine adult marmosets (Callithrix jacchus) contributed data to this study, aged 18-97 months

Wild animals

This study did not involve wild animals.

Reporting on sex

Only animals who were identified by the veterinary for humane euthanasia were used for this study, thus animals were not chosen based on sex. Six females were used, and three males.

Field-collected samples

This study did not involve field-collected samples.

Ethics oversight

Experimental procedures were approved by the University of Pittsburgh Institutional Animal Care and Use Committee.

Note that full information on the approval of the study protocol must also be provided in the manuscript.

Magnetic resonance imaging

Experimental design

Design type

Anatomical imaging

Design specifications

MPRAGE and FLASH sequences

Behavioral performance measures

Animals were anesthetized during anatomical imaging.

Acquisition

Imaging type(s)

Structural

Field strength

9.4 T

Sequence & imaging parameters

A T1-weighted fast low angle shot (FLASH) sequence was employed to detect the resultant shortening of T1 relaxation times from the contrast agents entering the parenchyma via the BBB disruption. Three scans (later averaged) were acquired for each animal with the following parameters: TR = 25 ms, TE = 8 ms, field of view = 35 x 35 x 26 mm, matrix size = 117 x 117 x 87, voxel size = 0.299 x 0.299 x 0.299 mm, bandwidth = 200 kHz, flip angle = 25 degrees, total scan time = 9 minutes, 2 seconds. For marmosets G & M, a magnetization prepared – rapid gradient echo (MPRAGE) sequence was used in lieu of the FLASH sequence because of the longitudinally-additive effects of systemic GBCA on the dynamic signal contrast. With the MPRAGE sequence, this additive effect is reduced with the additional inversion pulse. The MPRAGE sequence was acquired with the following parameters: TR = 6,000 ms, TE = 3.42 ms, field of view = 42 x

35 x 25 mm, matrix size = 168 x 140 x 100, voxel size = 0.250 x 0.250 x 0.250 mm, bandwidth = 50 kHz, flip angle = 14 degrees, total scan time = 20 minutes, 6 seconds.

Area of acquisition: Whole brain

Diffusion MRI: ☐ Used ☒ Not used

Preprocessing

Preprocessing software	FMRIB Software Library v6.0<br>AFNI Version AFNI_20.0.19 'Galba'<br>ANTs Version 5
Normalization	Data presented from individual animals, thus no normalization was necessary.
Normalization template	Data not normalized.
Noise and artifact removal	Raw MRI images clearly showed GBCA contrast, thus no processing was necessary.
Volume censoring	3D dataset, not 4D.

Statistical modeling & inference

Model type and settings	Anatomical data, 4D analyses not conducted.
Effect(s) tested	Anatomical data, 4D analyses not conducted.

Specify type of analysis: ☐ Whole brain ☐ ROI-based ☐ Both

Statistic type for inference<br>(See Eklund et al. 2016)	Anatomical data, 4D analyses not conducted.
Correction	Anatomical data, 4D analyses not conducted.

Models & analysis

n/a	Involved in the study
☒	☐ Functional and/or effective connectivity
☒	☐ Graph analysis
☒	☐ Multivariate modeling or predictive analysis
